# Telephone Counseling and Messaging Guided by Mobile Profiling of Tobacco Users for Smoking Cessation: A Randomized Clinical Trial

**DOI:** 10.1001/jamanetworkopen.2025.0764

**Published:** 2025-03-14

**Authors:** Yee Tak Derek Cheung, Min Jin Zhang, Tzu Tsun Luk, Sai Yin Ho, Tai Hing Lam, Man Ping Wang

**Affiliations:** 1 School of Nursing, Li Ka Shing Faculty of Medicine, The University of Hong Kong, Hong Kong SAR, China; 2 Alice Lee Centre for Nursing Studies, National University of Singapore, Singapore; 3 School of Public Health, Li Ka Shing Faculty of Medicine, The University of Hong Kong, Hong Kong SAR, China

## Abstract

**Importance:**

Evidence-based pharmacotherapy and behavioral therapy could increase tobacco abstinence but are underused and require further investigation.

**Objective:**

To evaluate the effectiveness of a novel mobile health (mHealth) profiling via ecological momentary assessment (EMA) in personalizing smoking cessation intervention for individuals who use tobacco with no intention to use smoking cessation services and medications.

**Design, Setting, and Participants:**

A 2-arm, assessor-blind, individual-randomized clinical trial was conducted. With online recruitment through social media or email advertisement, 459 adults with daily tobacco use in Hong Kong were recruited from March 23, 2022, to January 4, 2023, with follow-up completed by July 4, 2023. Individuals who had not used smoking cessation services or medications in the preceding 7 days and had no intention to use these aids in the next month were included. Participants were randomized 1:1 to intervention (n = 231) or control (n = 228) groups.

**Interventions:**

Both groups completed 5 EMAs daily for 7 days to report smoking behaviors and triggers. The intervention group additionally received nurse-led telephone counseling and 10-week instant messaging support guided by mHealth profiling from EMA.

**Main Outcomes and Measures:**

Primary outcomes were biochemically validated tobacco abstinence and Incremental Behavior Change Toward Smoking Cessation (IBC-S) score at 3 months after EMA initiation. Secondary outcomes were biochemically validated tobacco abstinence and IBC-S status at 6 months, self-reported 7-day point prevalence, abstinence, and use of smoking cessation medications and services at 3 and 6 months after EMA initiation. Intention-to-treat analysis was used.

**Results:**

The study included 459 participants (304 males [66.2%]) with a mean (SD) age of 36.7 (10.7) years. Biochemically validated tobacco abstinence rates were 8.2% in the intervention group and 3.5% in the control group (odds ratio [OR], 2.46; 95% CI, 1.06 to 5.75; *P* = .04) at 3-month follow-up. The corresponding rates at 6-month follow-up were 9.5% and 4.0% (OR, 2.56; 95% CI, 1.15-5.70; *P* = .02). The intervention group showed a greater increase in IBC-S scores from baseline to both 3-month (β = 1.03; 95% CI, 0.46-1.59; *P* < .001) and 6-month (β = 0.95; 95% CI, 0.37-1.53; *P* = .001) follow-ups.

**Conclusions and Relevance:**

The findings of this randomized clinical trial suggest that telephone counseling and instant messaging support, guided by mHealth profiling, increased tobacco abstinence vs EMA alone. This intervention may be used to supplement conventional smoking cessation for individuals who use tobacco and are unwilling to use smoking cessation aids.

**Trial Registration:**

ClinicalTrials.gov Identifier: NCT05212220

## Key Points

**Question:**

Is personalized smoking cessation intervention guided by mobile health profiling of ecological momentary assessment (EMA) effective for individuals who smoke with no intention to use smoking cessation aids?

**Findings:**

In this randomized clinical trial of 459 individuals who smoked daily, providing personalized nurse-led telephone counseling and instant messaging guided by mobile health profiling of EMA was associated with higher biochemically validated tobacco abstinence at 3 months compared with completing 7-day EMAs alone. The intervention group also had higher usage of smoking cessation aids compared with the control group.

**Meaning:**

Results of this study suggest that personalized telephone counseling and instant messaging guided by mobile health profiling of EMA can be used to supplement conventional smoking cessation promotion for individuals who use tobacco and are unwilling to use smoking cessation aids.

## Teaser

This randomized clinical trial evaluates the use of telephone counseling and instant messaging guided by mobile health profiling of individuals who smoke and are not using smoking cessation aids.

## Introduction

Tobacco use is a major modifiable risk factor for premature death,^1^ causing about 7.7 million deaths anually.^2^ Behavioral and pharmacologic interventions are safe and effective for smoking cessation,^1,3^ but their use remains low. Unassisted quitting is the predominant method for tobacco abstinence, with about two-thirds to three-quarters of individuals who smoke having attempted to quit unassisted worldwide.^4,5,6^ The World Health Organization recommends leveraging emerging technologies, such as mobile health (mHealth) to increase the accessibility of effective smoking cessation interventions.^3^

Tailored interventions based on individual smoking behaviors such as smoking triggers are common and effective smoking cessation interventions.^7,8,9,10^ Personalized telephone counseling increases tobacco abstinence (risk ratio, 1.38; 95% CI, 1.19-1.61) compared with self-help materials.^11^ Theory-based tailored text messaging could double the biochemical tobacco abstinence rate at 6-month follow-up compared with general messaging.^8^ However, lengthy profiling is not accepted for people who smoke who have no intention to seek smoking cessation aids. Mobile apps can facilitate real-time behavioral measurements and user profiling to guide tailored interventions.^12^ Nonetheless, about 90% of those apps require active long-term engagement from individuals who smoke,^13^ limiting the efficacy in those who do not seek smoking cessation aids. Therefore, there is a need for novel mHealth smoking cessation intervention that enables easy, low-intensity, and accurate smoking profiling to guide smoking cessation intervention.

Ecological momentary assessment (EMA) can prompt participants to report tobacco consumption, nicotine cravings, and smoking triggers by completing very short assessments at multiple time points in a day.^14,15^ Hence, it can be used as a profiling tool for guiding smoking cessation interventions, which is time-saving and highly accessible. In this randomized clinical trial (RCT), we assessed the effectiveness of a novel smoking cessation intervention, which used a self-developed EMA smartphone app to profile smoking behaviors, guiding personalized telephone counseling and instant messaging for tobacco users who had no intention to use smoking cessation aids.

## Methods

### Study Design

This was a 2-arm, open-label, RCT (randomization ratio 1:1). The original protocol approved by the institutional review board is available in Supplement 1, while a modified version was later published elsewhere.^16^ All participants completed 5 EMAs a day for 7 consecutive days to document smoking behaviors and smoking triggers. Participants were randomly assigned to 1 of the 2 conditions: (1) completion of 7-day EMAs with profiling and tailored interventions (intervention group) or (2) completion of 7-day EMAs only (control group). Both groups were followed up at 3 and 6 months after the EMA initiation. We followed the Consolidated Standards of Reporting Trials (CONSORT) reporting guideline. This study was approved by the institutional review board of the University of Hong Kong, Hospital Authority Hong Kong West Cluster. Informed consent was obtained from all participants before study enrollment. On completion of all 5 EMAs in 1 day, the participant received a $3.20 shopping voucher (all monetary values within the article are presented as USD), and an additional $3.20 shopping voucher if the overall EMA adherence rate was 80% or above. The total maximal incentive for completing all EMAs was $25.60.

### Participants

Participants were recruited both online and offline (eMethods in Supplement 2). Inclusion criteria were (1) age 18 years or older; (2) smoked at least 1 conventional cigarette, electronic cigarette, or heated tobacco product daily in the preceding 7 days, verified by salivary cotinine levels (≥30 ng/mL) (iScreen OFD Cotinine Saliva Test Kit; Abbott Toxicology); (3) owned a smartphone with internet access; (4) stayed in Hong Kong during the 7-day EMA; (5) were able to communicate in Chinese; (6) had no intention to use smoking cessation services or smoking cessation medications in the coming month; and (7) did not use any smoking cessation services and smoking cessation medications in the preceding 7 days. Individuals who were pregnant or had psychiatrist-diagnosed mental illness were excluded. Recruitment procedures were done remotely due to the COVID-19 pandemic (eMethods in Supplement 2).

### Randomization and Blinding

Randomization was conducted via the randomizer function of the survey platform (Qualtrics; Qualtrics Inc), where nonrecruitment staff entered their 5-digit identifiers, ensuring randomization concealment. Participants and interventionists could not be blinded, but outcome assessors were not aware of the group randomization at follow-up assessments. Data analysts were also blinded to the randomization until all prespecified analyses were completed.

### 7-Day EMA

All participants were instructed to download and set up our EMA App Smoking Radar (iOS or Android) during a virtual recruitment session via video call (WhatsApp or Zoom). Participants were prompted by the app’s push notifications to complete 5 fixed-interval signal-contingent EMAs a day for 7 consecutive days during waking hours (6-12 am), with a 3-hour gap between each EMA. Participants were required to report their tobacco consumption, nicotine craving, and tobacco products purchased in the past 3 hours, except for the first EMA each day, which was referred to as since the last EMA. They also reported triggers to smoking in 4 domains: withdrawal, emotional, social, and habitual (eFigure 1 in Supplement 2). Sleep quality and time to first tobacco product consumption of the day were also assessed every day at the first and second EMA (eMethods in Supplement 2).

### mHealth Profiling

After the 7-day EMA, our research assistant used the app’s back end to generate a report summarizing the smoking-related features, including (1) types of tobacco used, (2) tobacco consumption amount each day, (3) overall mean score of Heaviness of Smoking Index (HSI) during the 7-day EMA,^17^ (4) top 3 triggers of smoking, (5) top 3 triggers of nicotine craving, (6) top 3 triggers of purchasing tobacco, (7) number and types of craving (physical, psychological, and social), and (8) sleep quality (eMethods in Supplement 2). This information was used to guide the design of telephone counseling and instant messaging for the intervention group.

### Interventions

The intervention design was based on the theory of the Health Action Process Approach,^18^ which facilitates health behavior change in 2 phases: motivational phase, where individuals develop an intention to act, and volitional phase, involving the initiation and maintenance of the intended health behavior.^18^ In the motivational phase, the nurse-led telephone counseling aimed to raise awareness of personal smoking behaviors by summarizing the information from the mHealth profiling, including tobacco consumption amount each day, overall mean score of HSI, personal smoking triggers, and types of craving. In the volitional phase, the nurse motivated the participants to take action.

Participants were stratified by their willingness to quit stated at the baseline survey. For participants who were willing to quit, the quit plan included reasons to quit, set up a quit date (ideally within 2 weeks), strategies to overcome personal triggers, and referrals to smoking cessation services if required by the participants. For participants unwilling to quit, the nurse focused on increasing their motivation to quit by stating the relevant consequences of their tobacco use and nicotine dependence with reference from smoker profiling (eMethods in Supplement 2). The telephone counseling lasted about 20 minutes according to our intervention protocol.

Following the nurse-led telephone counseling, interventionists with at least 2 years of experience in smoking cessation research delivered 31 messages to participants in the intervention group over 10 weeks. The messages were sent on a tapering schedule from 8 in the first week to 1 in the last week. These messages were tailored to each participant’s smoking profile and delivered through instant messaging apps (eg, WhatsApp and WeChat). Messages included a profiling summary of smoking habits, HSI, triggers for smoking behavior, types of cravings, sex-specific harmfulness of smoking, benefits of quitting tailored by type of tobacco product used, and methods for managing nicotine withdrawal symptoms tailored by HSI and daily cigarette consumption (eTable 1 in Supplement 2). Additional messages included coping strategies for the top 3 triggers of smoking, the top 3 triggers of nicotine craving, and the top 3 triggers of purchasing tobacco. To increase participants’ engagement in the psychosocial support,^19^ participants could send messages to the interventionists anytime, but the interventionists responded only during office hours on working days.

### Outcomes

The primary outcomes were biochemically validated tobacco abstinence and behavioral progression toward quitting at 3-month follow-up. Tobacco abstinence was defined as no use of any tobacco products (including conventional cigarettes, heated tobacco products, electronic cigarettes) in the preceding 7 days, verified by exhaled carbon monoxide (Smokerlyzer, <4 ppm; Bedfont Scientific Ltd) and salivary cotinine (iScreen OFD Cotinine Saliva Test Kit, <30 ng/mL) (eMethods in Supplement 2). Individuals who self-reported quitting were invited for the validation in person. Behavioral progression was measured by Incremental Behavior Change Toward Smoking Cessation (IBC-S).^20^ The IBC-S has 2 subscales, including 12 items on behavioral changes (eg, I have picked a quit date: no = 0, yes = 1) and 3 items on cognitive changes (eg, I worry about how smoking is affecting my health) on a scale of 0 (never) to 4 (always). The higher IBC-S scores (score range, 0-24) indicate more readiness and preparation in quitting.

Secondary outcomes included biochemically validated tobacco abstinence and IBC-S scores at 6-month follow-up, self-reported 7-day point-prevalence abstinence, use of any smoking cessation service, and use of any smoking cessation medication at 3- and 6-month follow-ups. Other outcomes included quit attempts, which were measured by abstinence for at least 24 hours, and self-efficacy of quitting (eMethods in Supplement 2). Perceived helpfulness and satisfaction with the intervention were assessed at 3-month follow-up (eMethods in Supplement 2).

### Statistical Analysis

The sample size was calculated based on an anticipated intervention effect of an odds ratio (OR) of 2.92 obtained from a meta-analysis of tailored text messaging combined with counseling for smoking cessation^21^ and a biochemically validated tobacco abstinence of 4% among Hong Kong smokers receiving brief advice from another RCT.^10^ To detect a significant intervention effect with a power of 0.80 at a 2-tail significance level of α = .05, 222 participants per group were required (G∗Power software, version 3).

All analyses were done according to a prespecified analysis plan,^16^ with all analyses done using Stata/SE, version 16.1 (StataCorp LLC). A 2-sided *P* < .05 value indicated statistical significance. Intention-to-treat analysis was done by assuming no change in smoking behaviors from baseline for missing outcome measures. Logistic regression estimated the crude OR for biochemically validated abstinence, past 7-day point-prevalence abstinence, use of smoking cessation services, use of smoking cessation medications, and quit attempts at 3- and 6-month follow-ups. Linear regression estimated the crude regression coefficient (β) for IBC-S at 3-month follow-up. Linear mixed models with group × time interaction was used to examine the difference in change in IBC-S and self-efficacy from baseline to 3- and 6-month follow-ups. Multivariable regression and 2 prespecified sensitivity analyses, including multiple imputations and complete case analyses, were applied (eMethods in Supplement 2). A post hoc sensitivity analysis for the effectiveness of the intervention on primary and secondary outcomes by excluding 27 participants using only electronic cigarettes was also conducted. We used descriptive statistics for process evaluation variables within the intervention group. Additionally, we evaluated the cost-effectiveness of the new intervention by calculating the cost per additional participant who quit. We evaluated the consistency of intervention effect on the primary outcomes across subgroups by baseline characteristics and participants’ intervention engagement (eMethods in Supplement 2).

## Results

### Recruitment and Participants’ Characteristics

**Figure 1.**
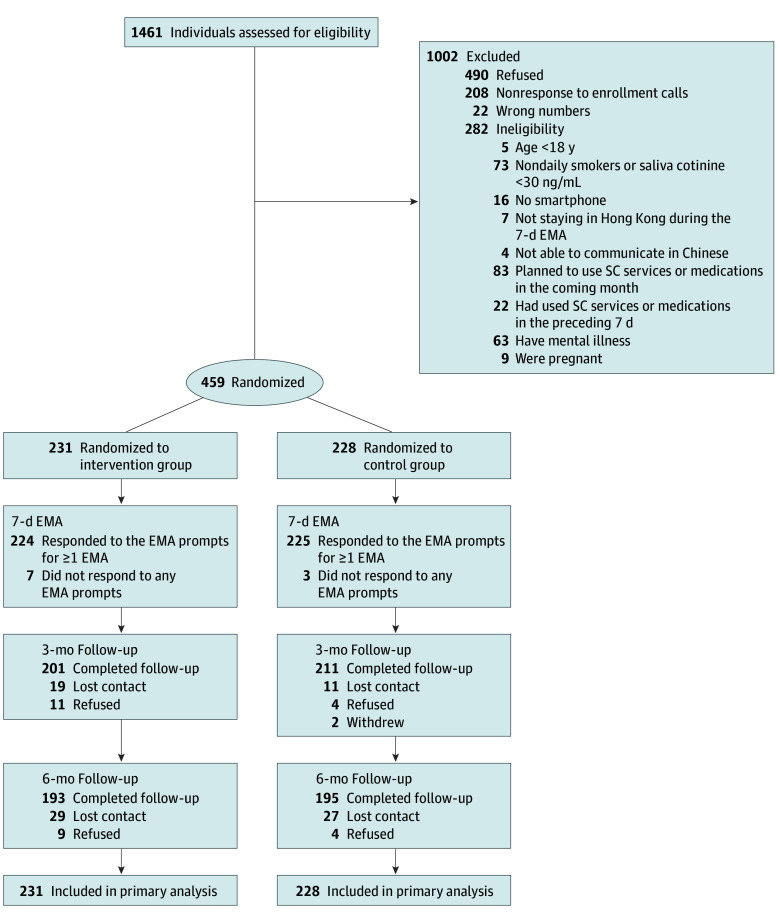
Trial Flowchart EMA indicates ecological momentary assessment; SC, smoking cessation.

From March 23, 2022, to January 4, 2023, 1461 potential participants were screened for eligibility, 1179 were eligible, and 459 participants (mean [SD] age, 36.7 [10.7] years; 304 males [66.2%]; 155 females [33.8%]) were randomized to the intervention (n = 231) or control (n = 228) groups (Figure 1). Retention rate was 89.8% at the 3-month follow-up, with no significant difference by trial groups. Younger age was associated with attrition at the 3-month follow-up (eTable 2 in Supplement 2). By intention-to-treat analysis, 459 participants were included in the primary analysis.

In all participants, 73.4% had no plan to quit, 63.4% had previously attempted to quit, and 44.9% had low levels of nicotine dependence. The mean (SD) IBC-S score for the intervention group was 6.1 (3.2) and the mean score for the control group was 6.0 (3.1) (Table 1).

**Table 1.** Sociodemographic and Smoking-Related Characteristics of the 459 Participants

Characteristic	Group, No. (%)	
	Intervention (n = 231)	Control (n = 228)
Age, mean (SD), y	36.0 (10.5)	37.4 (10.9)
Sex		
Male	147 (63.6)	157 (68.9)
Female	84 (36.4)	71 (31.1)
Educational level		
Primary or below	3 (1.3)	1 (0.5)
Secondary	108 (46.7)	107 (46.9)
Postsecondary	120 (52.0)	120 (52.6)
Monthly household income^a^		
<$3800	113 (49.4)	119 (52.4)
$3800-$7700	85 (37.1)	79 (34.8)
≥$7700	31 (13.5)	29 (12.8)
Type of tobacco product daily used^b^		
Conventional cigarettes	211 (91.3)	205 (89.9)
Heated tobacco products	21 (9.1)	22 (9.6)
Electronic cigarettes^c^	42 (18.2)	42 (18.4)
No. of cigarettes per day		
1-10	112 (43.9)	100 (48.5)
11-20	88 (38.1)	98 (43.0)
21-30	21 (9.1)	17 (7.5)
≥31	10 (4.3)	13 (5.7)
Nicotine dependence level (HSI score)^d^		
Low (0-2)	100 (43.3)	106 (46.5)
Moderate (3-4)	107 (46.4)	107 (46.9)
High (5-6)	24 (10.4)	15 (6.6)
Readiness to quit^a^		
Within 30 d	35 (15.2)	28 (12.3)
Within 6 mo	29 (12.6)	29 (12.7)
Beyond 6 mo, not decided yet, or no	166 (72.2)	171 (75.0)
IBC-S score, mean (SD)^a^^,^^e^	6.1 (3.2)	6.0 (3.1)
Previous quit attempts^a^		
No	78 (33.9)	89 (39.0)
Yes	152 (66.1)	139 (61.0)
Self-efficacy (score range, 0-10), mean (SD)		
Perceived importance to quit^a^^,^^f^	6.0 (3.0)	5.4 (3.1)
Perceived difficulty to quit^a^	7.2 (2.7)	7.1 (2.8)
Perceived confidence to quit^a^^,^^f^	4.9 (2.4)	4.4 (2.7)

Abbreviations: HSI, Heaviness of Smoking Index; IBC-S, Incremental Behavior Change Toward Smoking Cessation.

^a^ Data were not available for all participants.

^b^ Some participants were daily dual users of more than one type of tobacco product.

^c^ Twenty-seven participants exclusively used electronic cigarettes.

^d^ Score range 0 to 6, with a higher score indicating higher nicotine dependence; HSI is measured by 2 items: “How soon after you wake up do you smoke your first cigarette” (0 = >60 minutes, 1 = 31-60 minutes, 2 = 6-30 minutes, 3 = within 5 minutes) and “How many cigarettes per day do you usually smoke?” (0 = ≤10, 1 = 11-20, 2 = 21-30, 3 = ≥30).

^e^ Score range 0 to 24, with a higher score indicating more readiness and preparation in quitting.

^f^ *P* = .04 for reference only.

### Primary and Secondary Outcomes

**Figure 2.**
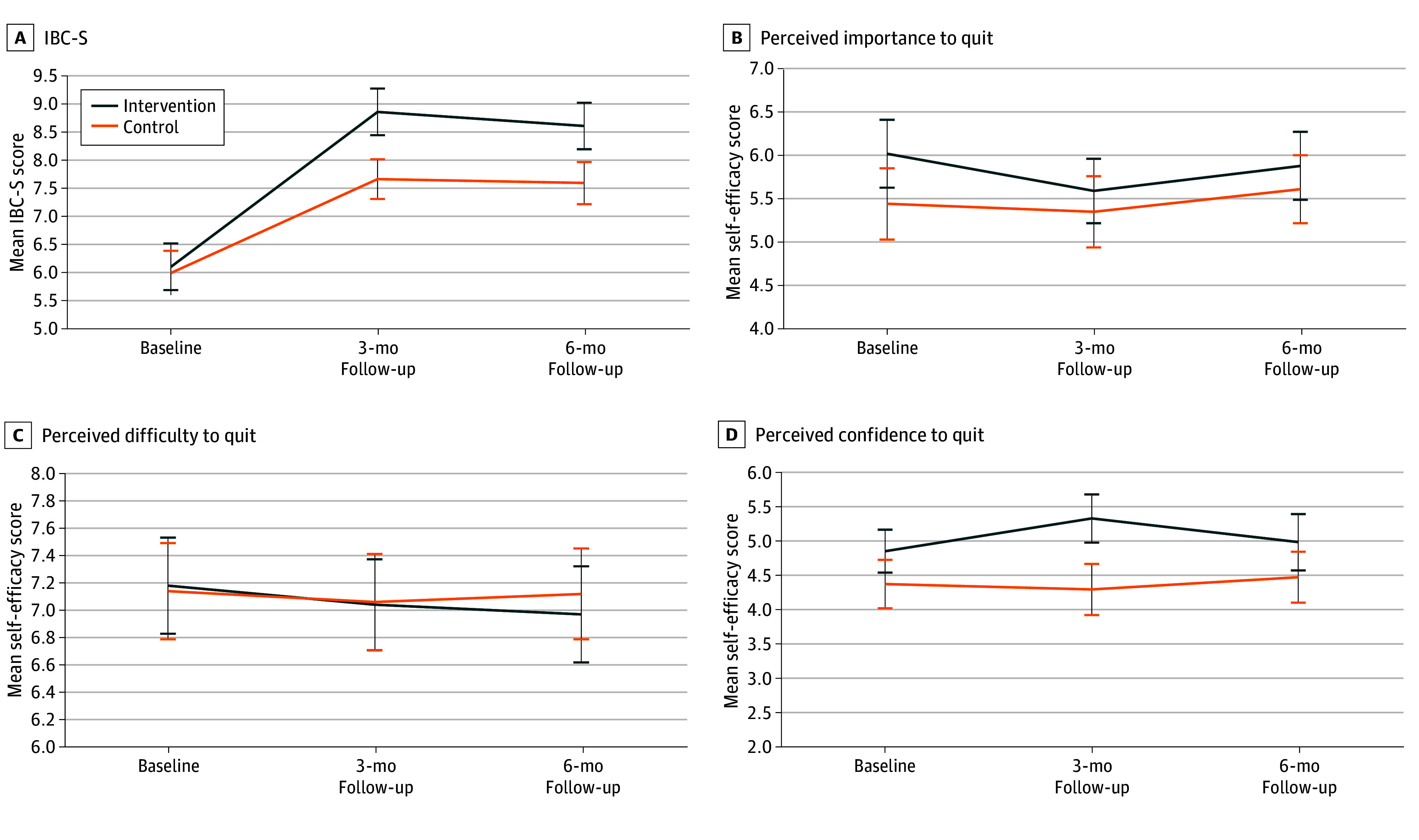
Differences in Change of Secondary and Other Outcomes From Baseline to 6-Month Follow-Up (N = 459) Group × time interactions. A, Incremental Behavior Change Toward Smoking Cessation (IBC-S), 3-month follow-up: regression coefficient (β) = 1.03; 95% CI, 0.46-1.59; *P* < .001; 6-month follow-up: β = 0.95; 95% CI, 0.37-1.53; *P* = .001. B, Perceived importance to quit: 3-month follow-up: β = −0.42; 95% CI, −0.93 to 0.08; *P* = .10; 6-month follow-up: β = −0.27; 95% CI, −0.78 to 0.25; *P* = .31. C, Perceived difficulty to quit: 3-month follow-up: β = −0.11; 95% CI, −0.53 to 0.31; *P* = .60; 6-month follow-up: β = −0.13; 95% CI, −0.56 to 0.30; *P* = .56. D, Perceived confidence to quit: 3-month follow-up: β = 0.57; 95% CI, 0.06-1.07; *P* = .03; 6-month follow-up: β = −0.03; 95% CI, −0.54 to 0.48; *P* = .92. Error bars indicate 95% CIs.

The participation rates of validation between the intervention and control groups at both 3-month (65.6% [21 of 32] vs 72.7% [8 of 11]; *P* = .67) and 6-month (71.0% [22 of 31] vs 58.8% [10 of 17]; *P* = .39) follow-ups were not significantly different. Table 2 reports the biochemically validated tobacco abstinence rate at 3-month follow-up was significantly higher in the intervention group than the control group (8.2% vs 3.5%; OR, 2.46; 95% CI, 1.06-5.75; *P* = .04), along with a significantly higher IBC-S score in the intervention group than the control group (β = 0.84; 95% CI, 0.30-1.38; *P* = .002). The intervention group also showed a higher rate of biochemically validated tobacco abstinence (OR, 2.56; 95% CI, 1.15-5.70; *P* = .02) at 6-month follow-up. The intervention group also reported significantly higher rates of 7-day point-prevalence abstinence PPA (3-month OR, 3.17; 95% CI, 1.56-6.46; *P* = .001; 6-month OR, 1.92; 95% CI, 1.03-3.58; *P* = .04), use of smoking cessation services (3-month OR, 7.72; 95% CI, 2.62-22.40; *P* < .001; 6-month OR, 6.61; 95% CI, 2.89-15.10; *P* < .001), and medication use (3-month OR, 5.83; 95% CI, 2.54-13.40; *P* < .001; 6-month OR, 6.29; 95% CI, 2.89-13.71; *P* < .001). Figure 2 shows that the intervention group had a significantly greater increase in IBC-S scores from baseline to 3-month (β = 1.03; 95% CI, 0.46-1.59; *P* < .001) and 6-month (β = 0.95; 95% CI, 0.37-1.53; *P* = .001) follow-ups.

**Table 2.** Primary, Secondary, and Other Outcomes

Outcome	Group		Logistic regression, OR (95% CI)/linear regression, β (95% CI)			
	Intervention (n = 231)	Control (n = 228)	Crude^a^	*P* value	Adjusted^b^	*P* value
**Primary outcomes**						
Biochemically validated tobacco abstinence at 3 mo, No. (%)	19 (8.2)	8 (3.5)	OR: 2.46 (1.06-5.75)	.04	OR: 2.73 (1.14-6.54)	.02
IBC-S score at 3 mo, mean (SD)	8.4 (3.2)	7.6 (2.7)	β: 0.84 (0.30-1.38)	.002	β: 0.81 (0.29-1.32)	.002
**Secondary outcomes, No. (%)**						
Biochemically validated tobacco abstinence at 6 mo	22 (9.5)	9 (4.0)	OR: 2.56 (1.15-5.70)	.02	OR: 3.16 (1.35-7.35)	.008
Self-reported 7-d point prevalence abstinence						
3 mo	32 (13.9)	11 (4.8)	OR: 3.17 (1.56-6.46)	.001	OR: 3.38 (1.64-7.00)	.001
6 mo	31 (13.4)	17 (7.5)	OR: 1.92 (1.03-3.58)	.04	OR: 2.26 (1.17-4.35)	.02
Use of smoking cessation services from baseline						
3 mo	28 (12.2)	4 (1.8)	OR: 7.72 (2.62-22.40)	<.001	OR: 8.31 (2.81-24.54)	<.001
6 mo (cumulative)	40 (17.3)	7 (3.1)	OR: 6.61 (2.89-15.10)	<.001	OR: 7.34 (3.15-17.12)	<.001
Use of smoking cessation medications from baseline						
3 mo	36 (15.6)	7 (3.1)	OR: 5.83 (2.54-13.40)	<.001	OR: 6.14 (2.62-14.40)	<.001
6 mo (cumulative)	43 (18.6)	8 (3.5)	OR: 6.29 (2.89-13.71)	<.001	OR: 6.71 (3.02-14.94)	<.001
**Other outcomes, No. (%)**						
Quit attempts from baseline^c^						
3 mo	58 (29.2)	46 (21.2)	OR: 1.53 (0.98-2.39)	.06	OR: 1.60 (0.99-2.58)	.05
6 mo (cumulative)	68 (36.4)	56 (27.1)	OR: 1.54 (1.01-2.36)	.05	OR: 1.60 (1.00-2.55)	.05

Abbreviations: β, regression coefficient; IBC-S, Incremental Behavior Change Toward Smoking Cessation; OR, odds ratio.

^a^ All analyses were done by logistic regression or linear regression.

^b^ Multivariable logistic and linear regressions adjusted for sex, age, nicotine dependence level (Heaviness of Smoking Index), readiness to quit, and previous quit attempts.

^c^ Excluded participants who self-reported quitting.

At the 3-month follow-up, differences in biochemically validated tobacco abstinence and IBC-S scores were significant across complete case analysis, analysis adjusting potential confounders, and analysis with multiple imputations (eTable 3 and eTable 4 in Supplement 2). The effect sizes for primary outcomes were robust after excluding 27 participants who exclusively used electronic cigarettes (eTable 5 and eTable 6 in Supplement 2).

Subgroup analyses for primary outcomes by sex, age, educational level, monthly household income, HSI, readiness to quit, previous quit attempts, and self-efficacy showed no significant interaction effect between group randomization and these characteristics (eFigure 2 and eFigure 3 in Supplement 2).

### Process Evaluation

All participants completed 75.3% (12 091 of 16 065) of the EMAs. The mean (SD) EMA adherence rates for the intervention and control groups were not significantly different (73.9% vs 76.7%, *P* = .19). A total of 96.1% of participants from the intervention group received nurse-led telephone counseling, and 97.4% received 10-week instant messaging support. Among those who received the nurse-led telephone counseling, 29.7% (66 of 222) requested a referral to current smoking cessation services and 25 of the referred participants reported attendance to these services at 6-month follow-up. The mean (SD) number of text messages posted by each intervention group participant was 7.3 (9.9).

eTable 7 in Supplement 2 reports that 9.0% of intervention group participants perceived the nurse-led telephone counseling as very helpful for increasing motivation to quit and 5.8% perceived it as very helpful for success in quitting. The corresponding proportions for 10-week instant messaging were 6.8% for increasing motivation and 3.7% as beneficial for success.

### Engagement Analyses

Table 3 reports that, in the intervention group, higher EMA adherence rates (adjusted OR, 1.04; 95% CI, 1.003-1.09; *P* = .04) and more responses to the 10-week instant messages (adjusted OR, 1.07; 95% CI, 1.03-1.12; *P* = .001) were associated with higher biochemically validated abstinence at the 3-month follow-up. More responses to the 10-week instant messages (adjusted β = 0.09; 95% CI, 0.05-0.13; *P* < .001) were also associated with a higher IBC-S score at the 3-month follow-up.

**Table 3.** Post Hoc Engagement Analyses of Biochemically Validated Tobacco Abstinence and IBC-S at 3-Month Follow-Up in the Intervention Group (N = 231)

Variable	Biochemically validated tobacco abstinence at 3 mo				IBC-S at 3 mo			
	Crude OR (95% CI)	*P* value	Adjusted OR (95% CI)^a^	*P* value	Crude β (95% CI)	*P* value	Adjusted β (95% CI)^b^	*P* value
EMA adherence rate^c^	1.04 (1.002 to 1.08)	.04	1.04 (1.003 to 1.09)	.04	0.01 (−0.01 to 0.03)	.23	0.01 (−0.01 to 0.03)	.16
No. of messages read by participants^d^								
None to half	1 [Reference]	NA	1 [Reference]	NA	NA	NA	NA	NA
Most of all to all	0.90 (0.47 to 2.49)	.84	0.99 (0.33 to 2.91)	.98	0.67 (−0.26 to 1.59)	.16	0.45 (−0.45 to 1.34)	.33
No. of responses posted by participants	1.05 (1.02 to 1.09)	.002	1.07 (1.03 to 1.12)	.001	0.11 (0.07 to 0.15)	<.001	0.09 (0.05 to 0.13)	<.001

Abbreviations: β, regression coefficient; EMA, ecological momentary assessment; IBC-S, Incremental Behavior Change Toward Smoking Cessation; NA, not applicable; OR, odds ratio.

^a^ Logistic regression adjusted for sex, age, nicotine dependence level (Heaviness of Smoking Index [HSI]), readiness to quit, and previous quit attempts.

^b^ Linear regression adjusted for sex, age, nicotine dependence level (HSI), readiness to quit, and previous quit attempts.

^c^ The proportion of completed EMAs by a participant compared with the total EMAs (n = 35).

^d^ In the intervention group who responded to 3-month follow-up (n = 201), 7 participants who self-reported not receiving the intervention and 1 participant who failed to respond to this question were excluded, resulting in a total of 193 participants included in the analysis.

### Cost-Effectiveness Analysis

The cost per individual who quit in the intervention group was $2051.3 and $3294.8 in the control group (eTable 9 in Supplement 2). The cost per additional individual who quit due to the intervention was $1147.87.

## Discussion

Our RCT has first shown that telephone counseling and instant messaging support, guided by mHealth profiling of smoking behaviors and triggers, were more effective than EMA alone in increasing biochemically validated abstinence and promoting readiness and preparation in quitting in individuals who use tobacco with no intention to use smoking cessation aids. We also found significant and large effect sizes in self-reported 7-day point-prevalence abstinence, use of smoking cessation services, and use of smoking medications at 3 and 6 months. Our post hoc analyses showed that, in the intervention group, higher EMA adherence rates and more responses to the 10-week instant messaging support were associated with higher biochemically validated tobacco abstinence at 3-month follow-up. Our intervention had similar cost per individual who quit with traditional behavioral support ($808)^22^ and financial incentives ($1716),^23^ which support that it provides good value for money.

Our RCT using mHealth profiling of full smoking features shows a greater effect size (OR, 2.46) than a single strategy, such as tailored text messages addressing limited baseline smoking characteristics reported in other studies (OR, 1.54; 95% CI, 1.19-2.00),^24^ telephone counseling (OR, 1.25; 95% CI, 1.15-1.35),^11^ and chat-based instant messaging support (OR, 1.69; 95% CI, 1.01-2.84).^10^ We propose 3 potential explanations. First, our mHealth profiling from EMA provided data in the real-world setting on tobacco consumption level, readiness to quit, withdrawal symptoms, tobacco purchase behavior, and smoking triggers with minimal recall bias, and facilitated a more personalized quit plan that could address specific smoking triggers. This benefit was corroborated by an association between EMA adherence rates and tobacco abstinence in the intervention group. Second, the nurse-led telephone counseling included a summary of smoking behaviors, quitting difficulties, and quit plans. These could have increased smokers’ preparation to quit and acceptance to adopt smoking cessation aids, thus subsequently promoted abstinence. At baseline, most of the participants were not ready to quit within 6 months, and none had an intention to use smoking cessation aids. Our mHealth profiling helped the participants accurately evaluate their nicotine dependence and quitting difficulties, and hence potentially increase their motivation to use smoking cessation aids. Also, the counseling effect for quitting and use of smoking cessation aids might be optimized when they had just completed the EMA with increased awareness of their smoking behavior. Third, after the telephone counseling, the 10-week instant messaging support could further increase or sustain participants’ motivation to quit and tobacco abstinence, which was supported by our process evaluation. Interacting with our cessation counselor could help participants better understand the instant messages and receive additional support during their quit attempts, thus increasing their tobacco abstinence. Our engagement analysis showed an association between the number of responses by participants in instant messaging and tobacco abstinence. Future secondary data analysis or qualitative study can examine the mediation effect of these factors to clarify the pathway.

Our findings have better generalizability due to 2 reasons. First, the biochemically validated abstinence at 3-month follow-up in our control group (3.5%), who only participated in the EMA, was similar to that of a previous pragmatic community-based cluster RCT on smoking cessation in Hong Kong in individuals who only received brief advice (4%).^10^ Although comparability of such results is uncertain, both studies were community-based trials targeting participants who were not seeking smoking cessation services. Therefore, the quit rate of the control group was close to that from minimum intervention. Second, 73.4% of our participants had no intention to quit, which was slightly higher than that of general Hong Kong current smokers (63.3%).^25^ Therefore, any bias due to self-selection of participants who opted for smoking cessation treatment should be low. An uncertain factor that may lower the clinical setting effect of our intervention is the provision of incentives to those who completed the EMA. In our RCT, incentives for EMA completion were required because participants needed compensation for their time to complete the EMAs, and the amount was modest. Future practice of mHealth profiling of smoking via EMA should consider including incentives to maintain high EMA adherence and collect sufficient information for mHealth profiling.

Our results have some implications for EMA and intervention development. First, our EMA design overcame low engagement by providing a simple interface, stable push notifications, and a flexible schedule for completing questionnaires, resulting in comparable EMA adherence rates to previous studies.^26^ Second, our intervention particularly fits for individuals who use tobacco who have barriers to participate in traditional smoking cessation services, such as a busy schedule or cost in taking time off work. Our flexible EMA schedule allowed participants to choose when to complete the EMA questionnaires. Third, the EMA app is a convenient profiling tool for personalized intervention that does not require tobacco users to receive face-to-face counseling in smoking cessation clinics or lengthy online self-assessments. Therefore, mHealth profiling can be developed as a new service model for smoking cessation.

### Limitations

Our study had some limitations. First, we targeted individuals who used tobacco with no intention to use smoking cessation aids. Whether our intervention is effective for those who are using smoking cessation services is uncertain, and future RCTs are warranted. Second, completing EMA might increase tobacco abstinence, as we found an association between EMA adherence rates and biochemically verified tobacco abstinence within the control group (eTable 8 in Supplement 2). We could not disentangle the individual effect of EMA, telephone counseling, and instant messaging support in this study due to the study design and insufficient sample size. Further factorial RCTs or secondary data analyses are needed to examine their individual effect. Third, EMA data accuracy might decrease due to response fatigue.^27^ Fourth, most participants had low to moderate tobacco dependence and consumed fewer than 20 cigarettes a day. Therefore, the intervention effect on heavy smokers is not certain. Fifth, this trial provided limited evidence on the mechanism underlying the intervention effect on tobacco abstinence. A qualitative study of participants’ perception of the EMA app and personalized interventions might provide some insight into these mechanisms.

## Conclusions

To our knowledge, this is the first RCT showing that nurse-led telephone counseling and instant messaging support, guided by mHealth profiling from EMA of smoking behaviors and smoking triggers, can at least double the odds of biochemically validated abstinence compared with EMA alone in individuals using tobacco who are unwilling to use smoking cessation aids. This intervention also increased the use of smoking cessation services and medications. These findings suggest that this intervention can be used to supplement conventional smoking cessation promotion for individuals using tobacco who are unwilling to use smoking cessation aids.

## Notes

Supplement 1.
Trial Protocol

Supplement 2. **eMethods.** Specific Details
eReferences
**eTable 1.** Example of Instant Messaging Guided by mHealth Profiling via EMA **eTable 2.** Baseline Characteristics by Follow-Up Status at 3-Month Follow-Up (N = 459) **eTable 3.** Sensitivity Analysis for Primary, Secondary, and Other Outcomes **eTable 4.** Multivariable Linear Mixed Models for Change of Secondary, and Other Outcomes From Baseline to 6-Month Follow-Up **eTable 5.** Primary, Secondary, and Other Outcomes, Excluding Participants Who Exclusively Used e-Cigarettes (N = 432) **eTable 6.** Change of Secondary and Other Outcomes From Baseline to 6-Month Follow-Up, Excluding Participants Who Exclusively Used e-Cigarettes (N = 432) **eTable 7.** Process Evaluation in the Intervention Group **eTable 8.** Association Between EMA Completion Rate With Primary, Secondary, and Other Outcomes in all Participants (N = 459) **eTable 9.** Costs of Conducting the Study Including Costs for Participants’ Recruitment, Incentives, EMA App Development, and Manpower **eFigure 1.** Description of Triggers for Smoking Behaviors (N = 12091) **eFigure 2.** Subgroup Analyses for Biochemically Validated Abstinence at 3-Month Follow-Up (N = 459) **eFigure 3.** Subgroup Analyses for IBC-S at 3-Month Follow-Up (N = 459)

Supplement 3.
Data Sharing Statement
