# Supplementary material for: Telephone Counseling and Messaging Guided by Mobile Profiling of Tobacco Users for Smoking Cessation: A Randomized Clinical Trial
Source: JAMA Netw Open. 2025 Mar 14;8(3):e250764. doi: 10.1001/jamanetworkopen.2025.0764 (PMC11909611; doi:10.1001/jamanetworkopen.2025.0764)
Supplement: Supplement 1. — Trial Protocol [file jamanetwopen-e250764-s001.pdf]

STUDY PROTOCOL

Project title: Low-intensity ecological momentary assessment (EMA) for smoking cessation intervention and tobacco control policy evaluation: A randomized controlled trial nested within an EMA-based observational study

1. Investigators

Principal investigator: Dr Yee Tak Derek Cheung, Research Assistant Professor, School of Nursing, HKU
Co-investigator: Prof. Tai Hing Lam, Chair Professor, School of Public Health, HKU
Co-investigator: Dr Man Ping Wang, Associate Professor, School of Nursing, HKU
Co-investigator: Dr William Ho Cheung Li, Associate Professor, School of Nursing, HKU
Co-investigator: Dr Vicky Wang, Post-doctoral Fellow, School of Nursing, HKU
Co-investigator: Ms Ching Han Helen Chan, Centre Supervisor, Tung Wah Group of Hospitals Integrated Centre on Smoking Cessation

2. Aims

(1) To evaluate the feasibility and efficacy of EMA-based smoking cessation intervention for smoking cessation
(2) To analyze the association between smoking cues and smoking-related behaviors in the local context. Smoking cues include exposure to POS tobacco displays, health warnings, scenes of others' smoking, alcohol use, social gathering, media items on promoting smoking cessation, feeling stressed/depressed, and other smoking-related cues. Smoking-related behaviors include purchase of tobacco, tobacco consumption, and nicotine craving.

3. Estimated duration and commencement data

Proposed starting date:	1 January 2021
Proposed study completion date:	31 December 2022
Expected final report date:	31 December 2022

4. Scientific / historical background

Tobacco control in Hong Kong Smoking is the leading cause of morbidity and pre-mature

mortality (1, 2). In Hong Kong, smoking kills 7,000 people each year (3), but there are still more than 620,000 daily smokers (4), half of whom will die prematurely due to smoking (5, 6). With stringent implementation of multi-pronged tobacco control policies in Hong Kong (7), the smoking prevalence has decreased by more than 50% from 23.3% in 1982 to 10.0% in 2017 (4). The government aims to further reduce the prevalence of adult current smoking to 7.8% by 2025 (8). However, the decreasing trend has slowed down since 2000 compared with that in the 1980s-2000 (4), and over half the smokers have no intention to quit (4). Therefore, new and effective strategies are urgently needed to achieve the single-digit target of smoking prevalence.

Gaps in smoking cessation intervention Smoking cessation services and prescription medications are either free or at very low out-of-pocket expense. Further, a telephone quitline is operated by the Department of Health. However, approximately 97% of current smokers have never used effective cessation aids, including counseling services and medication, in their quitting (4), with most quit attempters relying on self-determination (9). According to the Food and Health Bureau, only 25,745 smokers in 2016 availed smoking cessation services under Hospital Authority and services funded by the Department of Health, which is far below the number of quit attempters (113,800 in 2017) estimated by the Thematic Household Survey of Census & Statistics Department (4). Due to nicotine craving and environmental temptations, unaided quitting is very difficult (10). The low usage of current smoking cessation services and the preference of quit attempters suggest that simple interventions that support self-determination may meet the need of those who cannot afford the time for counselling or have low motivation for medications. Unfortunately, clinical trials on unassisted interventions (e.g., policy or low-intensity treatments) are scarce compared with those on assisted cessation intervention (e.g., medication or behavioral intervention) (11). Current smoking cessation services fail to support these smokers to quit and bring them to use effective cessation aids.

Gaps in tobacco control Key tobacco control policies (i.e., tobacco tax, extension of smoke-free areas, ban on tobacco advertising, and the new 85% health warnings on cigarette packs) have been implemented in Hong Kong, but more need to be done. Tobacco tax has not been raised for 5 years since the small increase of 11.7% in 2014, and cigarettes are now much cheaper in Hong Kong than in many developed countries. Seven countries have adopted tobacco plain packaging,

and at least seven (including Macau) countries/regions have banned tobacco displays in retail outlets. Also, widespread use of e-cigarettes and heated tobacco products risks renormalizing tobacco smoking as some users may eventually turn to combustible cigarettes. Unfortunately, the Hong Kong government has not yet passed new tobacco control measures in the above areas. Current cross-sectional studies (i.e., Thematic Household Survey and the Hong Kong Council on Smoking and Health [COSH] opinion survey) are insufficient to show causality between policy implementation and smoking behaviors, and hence new tobacco control measures are always criticized due to lack of local evidence. New research is needed to facilitate valid and accurate observations to inform and evaluate the effectiveness of tobacco control policies.

Ecological Momentary Assessment (EMA) EMA is a self-administered documentation of real-time data describing behavior, cognition, or event in the real world (12). EMA aims to understand "what is going on" close to the moment of assessment or over the very recent past; hence, such measurements have lower recall bias than traditional surveys (13) and are less subject to social desirability bias than reporting pervasive and negative behaviors over a longer period (14). EMA has the advantage of frequently collecting data at multiple time points in the natural environment, which is particularly useful to measure volatile additive behaviors and cognitions like tobacco use (12).

Three types of EMA have been commonly used for tobacco research (12). First, "event-contingent" assessment is used to yield data triggered by an event of interest and can be either initiated by subjects or automatically documented by sensors (e.g., Global Positioning System [GPS]). However, achieving high compliance of subject-initiated EMA is difficult as it relies on participants to actively input data and may pose large burden to the participants. Meanwhile, the accuracy of sensor-based EMA such as wristbands to recognize smoking and GPS is still emerging. The second type of EMA is "signal-contingent/time-based" assessment, which requires subjects to pre-schedule assessments at certain times or at random time in scheduled time blocks. The last type of EMA is to complete one retrospective assessment on previous behaviors and attitude at one particular time of a day (e.g., end-of-day [EoD] survey before night sleep). Time-based EMA and EoD survey will be applied in this proposed research because these assessments minimize subjects' burden and time, and the cost of instrument development is

lower.

EMA is a valid measurement tool to understand smokers' nicotine craving (15, 16) and environmental cues (17, 18). Measurements at different time points help clarify temporal and causal relationship between event exposures and smoking-related outcomes (12). For instance, EMA studies have revealed how nicotine craving is precipitated by internal stimuli like stress, depressed mood, negative affect, and pain. Another major research direction is to examine the effect of contextual factors on purchase and consumption of tobacco, such as tobacco marketing, health warnings, and alcohol use, using EMA data. Hence, the findings from EMA should have greater impacts on policy implementation than previous retrospective and cross-sectional studies.

Proposed research This research proposal is to make use of EMA for developing personalized smoking cessation interventions and for tobacco policy evaluation. As a policy evaluation tool, EMA allows more accurate data collection of real-world environment, subject to lower recall bias and social desirability bias. Another potential breakthrough is to develop a personalized quit plan and reminders based on EMA of each participant. EMA has been shown as a reliable instrument for documenting real-world smoking and quitting behaviors, which are useful for smoking cessation counselors to design a treatment plan incorporating medication and self-help behavioral interventions. A previous study showed the feasibility of delivering smoking cessation intervention using EMA (19), but no studies have evaluated the effectiveness of EMA-based and personalized interventions. Such EMA-based intervention protocol, if effective, will attract many more smokers to receive a personalized quit plan, without the need to attend multiple smoking cessation counseling sessions, and may motivate them to use effective cessation aids.

Previous EMA-based observational studies usually require participants to complete high-intensity EMA, which included at least 2-week EMA and 4 to 5 times of data collection on each day. These studies provided high amount (around US$80-230) of financial incentive to sustain an acceptable level of EMA. This EMA design may not be applicable in this proposed research, which aims to motivate smoking cessation but not only for observational study. We require sufficient real-world information about participants' smoking pattern for delivering personalized smoking cessation intervention. Prolonged data collection may reduce participants' motivation to

comply. Further, a high amount of incentive is not preferred as it may reduce the generalizability and induce bias in participation. Hence, we decided to develop a low-intensity EMA in this proposed research.

Pilot study I have conducted a pilot study (first in Asia) to examine the feasibility of using low-intensity EMA for documenting smoking cues and smoking-related behaviors (See additional materials). In total, 302 smokers installed the EMA app (designed by the primary investigator) in their own smartphone for a 2-day data collection and contributed 1,744 EMA (48% of all EMA prompts) and 693 valid EMA pairs for further analysis. The completion rate of 4+ and 6+ EMA pairs in the overall participants was 29.1% and 13.2%, respectively. Findings showed that exposure to tobacco point-of-sale (POS) displays (odds ratio [OR]=19.55, $p<.01$) and health warnings (OR=14.27, $p<0.02$) were significantly associated with subsequent purchase of tobacco, consistent with other EMA observational studies. Exposure to the POS displays apparently led to subsequent craving (OR=1.57) and tobacco consumption (OR=1.47), but the sample size was not sufficient to show statistical significance. Findings were presented in the International Symposium on Digital Health 2019 (20).

During Jan 2020 to Oct 2020, I conducted another pilot study using EMA to investigate exposure to alcohol advertisements and alcohol drinking culture in university students. Participants completed 5 regularly prompted EMA surveys with an EMA app in their smartphone every day for 14 consecutive days. The results showed that the 48 participants responded in 2474 EMA (73.5% of all EMA prompts). The compliance rate of 49+ (70%) and 56+ (80%) EMA was 70.8% and 45.8%, respectively.

These two pilot studies suggests several improvements to increase the EMA compliance: (1) Complicated steps to set up an EMA schedule with the EMA app increase participants' burden in real-world participation. Future app development should reduce these complicated steps and use a more user-friendly interface. A simpler set-up procedure can also save recruitment time for each participant in the outdoor environment, as their available time for recruitment is limited. (2) To sustain a higher EMA compliance for time-based system-triggered EMA, separate assessments (in the alcohol EMA pilot study) is more preferred than pair-based assessments (in the tobacco pilot

EMA). Meanwhile, a cumulative incentive was efficient in improving the compliance rate. (3) Higher but manageable intensity for EMA is needed. Smoking cessation nurses need additional smoking information (e.g., influence from peer or family, stress, depressed mood) to assess nicotine dependency and specific smoking-related cues. Also, more days of EMA may increase the chance of capturing infrequent contextual exposures. Therefore, the days of time-based EMA will be increased from 2 to 7 days. EoD surveys will also be included for assessing daily tobacco consumption throughout a day.

5. Hypothesis

We hypothesize that smokers who receive the EMA-based smoking cessation intervention will have higher scores in Incremental Behavior Change towards Smoking Cessation (IBC-S), which measures the behavioral progression towards smoking cessation, and higher biochemically validated abstinence than those who do not receive the intervention at 3-month follow-up.

6. Study design

The proposed study comprises a 2-arm (allocation ratio 1:1) pragmatic RCT to assess the efficacy of the EMA-based smoking cessation intervention and the EMA-based observational study. Throughout the 7-day participation, all recruited participants will complete an EoD survey and 4 time-based system-triggered EMA each day. The surveys will be prompted by a smartphone application (app) installed in their own mobile phones. The prompting time will be personalized and pre-scheduled by the participants. The information collected in the app will be used to develop an EMA-based intervention including a quit plan and 10-week quitting reminders and for an EMA-based observational study. I will randomly allocate half of the participants (intervention group) to receive the EMA-based intervention. All participants will then be contacted for a follow-up at 3-month and 6-month.

7. Subjects

This proposed study aims to recruit daily tobacco users, regardless of intention to quit. We shall send mass emails to the University of Hong Kong (HKU) staff and students, post recruitment advertisements in media and smoking cessation clinics, or actively invite smokers at outdoor smoking hotspots to participate. Outdoor smoking hotspots (i.e., urban places with a butt

collector and a cluster of smokers) were shown as feasible platforms for efficient smokers' recruitment (21). The inclusion criteria are: (1) daily consumption of tobacco products (including traditional cigarettes, electronic cigarettes, and heated tobacco products) in the past week; (2) age ≥18 years; (3) owning a mobile smartphone with internet access; (4) staying in Hong Kong during the 1-week EMA study period; and (5) able to read and write Chinese. To confirm the smoking status of the participants, exhaled carbon monoxide will be measured and required to be 4ppm or above, or saliva cotinine to be 30ng/ml or above. Because the proposed study aims to promote smoking cessation in smokers who do not plan to use smoking cessation aids, we will exclude smokers who (1) plan to use smoking cessation services or medication in the coming month or (2) using smoking cessation services, or using nicotine replacement therapy in the past 7 days. Smokers who reported having mental illnesses and female smokers who are pregnant are also excluded as they need more specific smoking cessation intervention.

To date, there have been no randomized controlled trial (RCT) to provide an estimate of effect size for the EMA-based intervention. Our team's previous RCT of testing a personalized smoking cessation intervention showed that the effect size (risk ratio) can be as high as 1.68 (22). Our current outreach health promotion (funded by Health Care Promotion Scheme, project no. 01170418) showed that each month we can recruit 30 to 60 smokers to participate in the smoking cessation RCT. Considering the limited study period covered in this funding application, this proposed study targets to recruit 440 smokers in 11 months.

8. Recruitment procedures

In the outdoor recruitment sessions, the recruitment staff will approach and distribute souvenirs (e.g., a tissue pack) or leaflets to the smokers at outdoor smoking hotspots for 3-4 hours in daytime. If a smoker is willing to accept them and talk to the recruitment staff, the staff will advise the smoker to quit using the AWARD protocol and ask further questions related to eligibility. The AWARD protocol includes (1) **A**sk the smoking history, (2) **W**arn about the high risk (i.e., half of the smokers will die of smoking-related diseases), (3) **A**dvise to quit, (4) Introduce smoking cessation services and medications (**R**efer), and (5) Repeat the above advice (**D**o-it-again) (23). Our previous studies have shown that the AWARD protocol is a feasible and appropriate tool used by non-healthcare professionals to promote smoking cessation.

The promotion sessions will be organized at outdoor smoking hotspots, which will be selected from the public places in our funded project of Health Care Promotion Scheme (Project number 01170418). These locations cover New Territories, Kowloon, and Hong Kong Island, and the feasibility study supports that the number of subjects recruited in these locations will be higher than that in other places. Thus, they will be used in this study. Pre-promotion site visits and replacement of hotspots with reduced number of smokers will be done to ensure that the maximum number of smokers can be approached.

Eligible participants will be given more details of the study, and asked to consent to participation via an online form on Qualtrics. Then, the staff will assist the participants to install the EMA app on their mobile phones. Participants will need to complete a simple baseline questionnaire, which documents sociodemographic characteristics, smoking cues, smoking behaviours and contact information. Afterwards, the staff will ask the participants to select 7 consecutive days as the study period. Participants will be asked to set up the time they would like to receive the first time-based EMA from 6:00 am to 12:00 nn to undertake the regularly prompting EMA. For example, if a participant set the first prompt time at 9:00 am, he will be asked to complete an EMA survey at 9:00 am, 12:00 nn, 3:00 pm, 6:00 pm and an EoD survey at 9:00 pm. Then the staff will set up the EMA schedule in the app for the participants.

Because the recruitment time for each participant at outdoor hotspots is always limited, recruitment procedures will be brief. Participants need not set up the EMA schedule with the app at recruitment. Instead, the staff will document the EMA schedule and proceed with the set-up procedures in the back-end after the recruitment session. A pocket-sized card including the EMA schedule and instruction of using the app will be provided onsite.

Some participants will know the study from publicity emails or advertisement. If they are interested, they can complete an online application form (using Qualtrics or Google form). Recruitment staff will make appointment with the interested participants to complete the recruitment procedures.

Our recruitment staff will provide virtual recruitment procedures for those potential participants who don't like to have face-to-face recruitment procedures. First, the recruitment staff will contact those participants and delivery a cotinine saliva test device to the potential participants. Then the recruitment staff will have a virtual meeting with the potential participants after they receive the cotinine saliva test device. During the virtual meeting, our recruitment staff will guide the potential participants to validate their cotinine level, then assist eligible participants in completing the baseline questionnaire, and install and set the EMA app.

9. Randomization and allocation concealment

Simple individual randomization (1:1 allocation ratio) will be applied. Qualtrics' randomizer will be used for random allocation. This will be implemented by a research staff, who is not involved in the recruitment, so that all recruitment staff and participants will be concealed to the group allocation at recruitment. After a participant consent to the trial, our research staff will access the randomizer in Qualtrics and enter the individual participant's 5-digit identifier. With the randomizer feature in place, Qualtrics will display a page indicating either the intervention group or the control group. A separate Qualtrics account for the randomizer will be used, and group allocation will be separated from research data. Information of the participants in the intervention group will be transferred to the research nurse for the EMA-based intervention.

10. Blinding

Participants will not be blinded to the intervention. Assessors of the follow-up outcomes and the research investigators will not be involved in the recruitment and intervention delivery and will be blinded to the group allocation (single-blinded design).

11. Intervention

Smartphone application A EMA app will be developed to facilitate documentation of all smoking cues and smoking-related behaviors. After each recruitment, the staff will set up all EMA schedules of the participants in the back-end. Participants can then receive the EMA prompts and complete the EMAs. Participants will use the last 5 digits of their mobile phone numbers as identifier. They will use the app to complete an EoD survey and 4 time-based EMA each day in the 7 consecutive study days.

In each time-based EMA time window, the app will firstly prompt the user to answer questions on the smoking cues, smoking behaviours , and the association between smoking cues and smoking-related behavior, which only takes about 1 minute. If the user does not respond to an EMA prompt, 2 subsequent prompts will be generated. If these additional prompts are not responded to, the EMA will regard nonresponse as missing data. All data will be uploaded to our server immediately after each EMA completion.

The EMA questions on smoking cues cover 3 domains: mood, sleep, environmental triggers. The mood domain includes (1) irritable, depressed, restless, tension, stress, and other emotional cues related to smoking; Sleep domain includes insomnia symptoms for the last night. Environmental triggers include (1) social gathering; (2) family/friends/partner smoking; receive tobacco products/encouragement from others; (3) any other environmental smoking cues. Smoking-related behaviors include (1) nicotine craving (2) whether he/she has bought/consumed tobacco (traditional cigarettes, heated tobacco products and electronic cigarettes) in the past 3 hours (yes/no), (3) whether the tobacco consumption has been due to the exposure to the pro-smoking cues, and (4) other reasons of tobacco consumption (e.g., kill time, habit, leaving a place where smoking is not allowed). EoD survey includes (1) daily tobacco consumption; (2) time to consume the first cigarette after waking; (3) all EMA questions; (4) tobacco control policy questions which include daily exposure to health warnings on their tobacco pack/POS tobacco display/exposure to hotspot.

Participants will be encouraged to improve compliance via WhatsApp reminders if their compliance rate drops to 50% or below in the first 3 study days. To increase uptake and compensate the time spent by the participants, a small amount of cash incentive is needed. All participants will be given a HK$50 gift voucher for the completion of baseline survey. They will be rewarded a HK$ 15 gift voucher for completing 3 time-based EMA surveys within each day. They will be further rewarded a HK$ 10 gift voucher for completing all surveys in each day. In addition, participants will be further rewarded HK$ 25 gift voucher for completing more than 80% of 7 days time-based EMA surveys. Participants who complete biochemical validation at 3 months or 6 months follow up will obtain an additional HK$50 gift voucher.

EMA-based intervention Based on the EMA of each participant, a trained research nurse will review their (1) nicotine dependence; (2) intensity and frequency of reported cravings; and (3) the impact of exposure to pro-smoking cues on smoking behaviors. A personalized quit plan will be developed based on tailored suggestions form the US clinical practice guideline for smoking cessation, Smoking Cessation, Smoking Cessation Information Kit (published by Department of Health), the theory of Health Action Process Approach, and the self-determination theory. Our recent studies showed that interventions based on these theories are effective (22, 24). Half of the randomly selected participants (intervention group) will receive this quit plan via a nurse-led phone call, email, and WhatsApp. The nurse will then design a quit plan including (1) self-help techniques on handling craving; (2) suggestions to avoid the reported pro-smoking cues; (3) whether to quit progressively or abruptly; (4) referral for existing smoking cessation service; and (5) instruction of using over-the-counter (OTC) nicotine replacement therapy (NRT), if OTC-NRT is preferred by the participant. Participants will then receive tailored quitting reminders via instant messaging social media (i.e.,WhatsApp, WeChat) and voluntarily discuss their quit plan with the nurse in the subsequent 10 weeks.

12. Main outcome measures

The primary outcome measures are changes in the 15-item Incremental Behavior Change towards Smoking Cessation (IBC-S) (25), which detects behavioral progression towards smoking cessation, and tobacco abstinence in the past 7 days at 3-month follow-up, which is validated using exhaled carbon monoxide (<4 ppm) and salivary cotinine (<30 ng/ml). The secondary outcome measure are (1) self-reported use of smoking cessation service or medication since baseline at 3 and 6-month follow-up; (2) self-report 7-day abstinence at 3 and 6-month follow-up; (3) biochemical validated abstinence at 6-month follow-up. Feasibility measures include the satisfaction score towards the intervention and the EMA compliance.

13. Follow-up

All participants will be followed up via telephone by an allocation-blinded interviewer at 3 and 6 months after consenting to participation. Only the participants who report abstinence in the past 7 days will be invited for measurement of their exhaled CO with a Smokerlyzer and salivary

cotinine with a cotinine saliva test device at their residence, workplace, or nearby, according to their preference. To increase participation, the participants will be given HK$50 (approximately equivalent to US$6.4) as a compensation for their time cost. All participants will not know the validation incentive at baseline. The validation takes a very short time (15 seconds) and is easy. Participants' satisfaction toward EMA app and EMA questionnaire will be assessed via telephone one week after the compliance of the EMA period. The satisfaction toward EMA will be evaluated by the nurse-phone call for the intervention group and by our allocation-blinded interviewer for the control group.

14. Statistical analysis

Intention-to-treat approach will be used to include all consented participants in the analysis and assume non-respondents at the 3-month follow-up as smokers. Chi-squared and t-tests will be used to compare sociodemographic and smoking characteristics of participants to assess the balance between intervention and control group.

The primary comparison is the prevalence of tobacco abstinence and the score of IBC-S between the EMA-based smoking intervention group and the control group. Logistic regression model and linear regression will be used to compare the 7-day PPA and the score of IBC-S between intervention and control group at 3 months, respectively. We will also use multiple logistic regression and multiple linear regression adjusted for imbalance socio-demographic smoking characteristics assessed at baseline to account for imbalances in baseline characteristics.

We will transfer checklist options of smoking cues to binary outcomes. For example, an EMA question asked 'In the past 3 hours, have you been exposed to (check all that apply" followed by these checklist options: family smoking; friends smoking; partner smoking; receive tobacco products from others; receive encourage of smoking from others). We will recode response into a series of dichotomous variables. Generalized estimating equation analysis will be used to examine the causal relationship between smoking cues and smoking-related behaviors in all participants. A linear mixed model will be used for the association analysis when the outcome is not binary.

Direct operating costs will include app development, salary (of nurses and research assistants), recruitment, telephone follow-up, and publicity items. Costs for study design, analysis, report

writing, and irrelevant administration will be excluded. The cost per successfully recruiting a smoker to participate will be calculated by dividing the total cost by the number of smokers who have joined. Similarly, cost per participant who use smoking cessation aids and per quitter at 3-month follow-up will be calculated.

15. Consent

Participation in the study is voluntary. The recruitment staff will first explain the project's details to the potential subjects who agree to join the study before seeking consent. The potential subjects will be assured that they can withdraw from the study anytime without any prejudice, and all the information will be kept confidential and results will be reported in an aggregate format. After that, subjects will be directed to an online form on Qualtrics to consent to participation.

16. Ethics

Ethics approval will be sought from the Institutional Review Board of the University of Hong Kong / Hong Kong Hospital Authority Hong Kong West Cluster.

17. Direct access to source data/documents

The EMA mobile App will only request participants to enter research data, and a 5-digit case number will be used for necessary identification. The raw data from the baseline survey, EMA App and post-EMA survey will be stored in an external hard-disk and locked in a cupboard with keys kept by the Principal Investigator. Only the investigators and research assistants of the project will be permitted to access the raw data and/ or study records. The data will be scanned and kept for 10 years or longer after the study is completed. Individual participants will not be directly identifiable from the dataset to be used for analysis.

18. Chronological outline of research plan

<table>
<tr><th>Year</th><th colspan="12">2021</th><th colspan="12">2022</th></tr>
<tr><th>Month</th><th>1</th><th>2</th><th>3</th><th>4</th><th>5</th><th>6</th><th>7</th><th>8</th><th>9</th><th>10</th><th>11</th><th>12</th><th>1</th><th>2</th><th>3</th><th>4</th><th>5</th><th>6</th><th>7</th><th>8</th><th>9</th><th>10</th><th>11</th><th>12</th></tr>
<tr><td>Development of the instrument and protocol</td><td style="background:#c0c0c0"></td><td style="background:#c0c0c0"></td><td></td><td></td><td></td><td></td><td></td><td></td><td></td><td></td><td></td><td></td><td></td><td></td><td></td><td></td><td></td><td></td><td></td><td></td><td></td><td></td><td></td><td></td></tr>
<tr><td>EMA App production</td><td style="background:#c0c0c0"></td><td style="background:#c0c0c0"></td><td style="background:#c0c0c0"></td><td style="background:#c0c0c0"></td><td style="background:#c0c0c0"></td><td style="background:#c0c0c0"></td><td style="background:#c0c0c0"></td><td style="background:#c0c0c0"></td><td></td><td></td><td></td><td></td><td></td><td></td><td></td><td></td><td></td><td></td><td></td><td></td><td></td><td></td><td></td><td></td></tr>
<tr><td>Subjects recruitment and data collection</td><td></td><td></td><td></td><td></td><td></td><td></td><td></td><td></td><td style="background:#c0c0c0"></td><td style="background:#c0c0c0"></td><td style="background:#c0c0c0"></td><td style="background:#c0c0c0"></td><td style="background:#c0c0c0"></td><td style="background:#c0c0c0"></td><td style="background:#c0c0c0"></td><td style="background:#c0c0c0"></td><td style="background:#c0c0c0"></td><td style="background:#c0c0c0"></td><td style="background:#c0c0c0"></td><td style="background:#c0c0c0"></td><td></td><td></td><td></td><td></td></tr>
<tr><td>Data analysis</td><td></td><td></td><td></td><td></td><td></td><td></td><td></td><td></td><td></td><td></td><td></td><td></td><td></td><td></td><td></td><td></td><td></td><td></td><td></td><td></td><td style="background:#c0c0c0"></td><td style="background:#c0c0c0"></td><td style="background:#c0c0c0"></td><td style="background:#c0c0c0"></td></tr>
<tr><td>Report writing</td><td></td><td></td><td></td><td></td><td></td><td></td><td></td><td></td><td></td><td></td><td></td><td></td><td></td><td></td><td></td><td></td><td></td><td></td><td></td><td></td><td style="background:#c0c0c0"></td><td style="background:#c0c0c0"></td><td style="background:#c0c0c0"></td><td style="background:#c0c0c0"></td></tr>
</table>

19. Deliverables

The initial findings will be submitted for application for the coming round of the Health Medical Research Fund. The findings will be disseminated through publication in the journal Addiction or Addictive Behaviour.

20. Dissemination of study result

The research findings will be disseminated in local and international conferences related to public health or substance use.

21. Financing and insurance

Research Fund: Health Medical Research Fund Research Fellowship Scheme

22. References

1. Forouzanfar M. H., Alexander L., Anderson H. R., Bachman V. F., Biryukov S., Brauer M. et al. Global, regional, and national comparative risk assessment of 79 behavioural, environmental and occupational, and metabolic risks or clusters of risks in 188 countries, 1990–2013: a systematic analysis for the Global Burden of Disease Study 2013, The Lancet 2015: 386: 2287-2323.
2. Gakidou E., Afshin A., Abajobir A. A., Abate K. H., Abbafati C., Abbas K. M. et al. Global, regional, and national comparative risk assessment of 84 behavioural, environmental and occupational, and metabolic risks or clusters of risks, 1990–2016: a systematic analysis for the Global Burden of Disease Study 2016, The Lancet 2017: 390: 1345-1422.
3. Mcghee S., Ho L., Lapsley H., Chau J., Cheung W., Ho S. et al. Cost of tobacco-related diseases, including passive smoking, in Hong Kong, Tob Control 2006: 15: 125-130.
4. Census & Statistics Department (Hong Kong Sar Government). Thematic Household Survey, Report No. 64: Pattern of Smoking, Hong Kong: Census & Statistics Department 2018.
5. World Health Organization. A guide for tobacco users to quit, Geneva: World Health Organization; 2014.
6. Lam T. H. Absolute risk of tobacco deaths: One in two smokers will be killed by smoking: comment on “smoking and all-cause mortality in older people”, Arch Intern Med 2012: 172: 845-846.
7. Koplan J. P., An W. K., Lam R. M. K. Hong Kong: a model of successful tobacco control in China, The Lancet 2010: 375: 1330-1331.
8. Food and Health Bureau, Department of Health. Towards 2025: Strategy and action plan to prevent and control non-communicable diseases in Hong Kong, Hong Kong: Department of Health; 2018.
9. Smith A. L., Chapman S., Dunlop S. M. What do we know about unassisted smoking cessation in Australia? A systematic review, 2005–2012, Tob Control 2015: 24: 18-27.
10. Borland R., Li L., Driezen P., Wilson N., Hammond D., Thompson M. E. et al. Cessation assistance reported by smokers in 15 countries participating in the International Tobacco Control (ITC) policy evaluation surveys, Addiction 2012: 107: 197-205.

11. Chapman S., Mackenzie R. The Global Research Neglect of Unassisted Smoking Cessation: Causes and Consequences, PLoS Med 2010: 7: e1000216.
12. Shiffman S. Conceptualizing Analyses of Ecological Momentary Assessment Data, Nicotine Tob Res 2014: 16: S76-S87.
13. Schwarz N. Retrospective and concurrent self-reports: the rationale for real-time data capture. In: Stone A. A., Shiffman S., Atienza A. A. & Nebeling L., editors. The science of real-time data capture: Self-reports in health research, New York: Oxford University Press; 2007, p. 11-26.
14. Collins R. L., Martino S. C., Kovalchik S. A., Becker K. M., Shadel W. G., D'amico E. J. Alcohol Advertising Exposure Among Middle School–Age Youth: An Assessment Across All Media and Venues, Journal of Studies on Alcohol and Drugs 2016: 77: 384-392.
15. Serre F., Fatseas M., Swendsen J., Auriacombe M. Ecological momentary assessment in the investigation of craving and substance use in daily life: A systematic review, Drug and Alcohol Dependence 2015: 148: 1-20.
16. Bolt D. M., Piper M. E., Theobald W. E., Baker T. B. Why two smoking cessation agents work better than one: role of craving suppression, J Consult Clin Psychol 2012: 80: 54-65.
17. Watkins K. L., Regan S. D., Nguyen N., Businelle M. S., Kendzor D. E., Lam C. et al. Advancing Cessation Research by Integrating EMA and Geospatial Methodologies: Associations Between Tobacco Retail Outlets and Real-time Smoking Urges During a Quit Attempt, Nicotine Tob Res 2014: 16: S93-S101.
18. Roberts M. E., Keller-Hamilton B., Hinton A., Browning C. R., Slater M. D., Xi W. et al. The magnitude and impact of tobacco marketing exposure in adolescents' day-to-day lives: An ecological momentary assessment (EMA) study, Addict Behav 2019: 88: 144-149.
19. Businelle M. S., Ma P., Kendzor D. E., Frank S. G., Vidrine D. J., Wetter D. W. An Ecological Momentary Intervention for Smoking Cessation: Evaluation of Feasibility and Effectiveness, J Med Internet Res 2016: 18: e321.
20. Cheung Y. T. D., Lam T. H., Chan C. H. H., Ho K. S., Fok W. Y. P., Wang M. P. et al. Ecological momentary assessment for the evaluation of tobacco health warnings, point-of-sale tobacco displays and smoking hotspots. International Symposium on Digital Health 2019, Hong Kong; 2019.
21. Cheung Y. T. D., Lam T. H., Li W. H. C., Wang M. P., Chan S. S. C. Feasibility, Efficacy and Cost Analysis of Promoting Smoking Cessation at Outdoor Smoking "hotspots": a pre-post Study, Nicotine Tob Res 2018: 20: 1519-1524.
22. Wang M. P., Luk T. T., Wu Y., Li W. H., Cheung D. Y., Kwong A. C. et al. Chat-based instant messaging support integrated with brief interventions for smoking cessation: a community-based, pragmatic, cluster-randomised controlled trial, The Lancet Digital Health 2019: 1: e183-e192.
23. Chan S. S. C., Wong D. C. N., Cheung Y. T. D., Leung D. Y. P., Lau L., Lai V. et al. A block randomized controlled trial of a brief smoking cessation counselling and advice through short message service on participants who joined the Quit to Win Contest in Hong Kong, Health Educ Res 2015: 30: 609-621.
24. Li W. H. C., Ho K. Y., Wang M. P., Cheung D. Y. T., Lam K. K. W., Xia W. et al. Effectiveness of a brief self-determination theory-based smoking cessation (immediate or progressive) intervention for smokers attending emergency departments: a randomised controlled trial, JAMA Intern Med 2019: In press.
25. Flocke S. A., Step M. M., Lawson P. J., Smith S., Zyzanski S. J. Development of a Measure of Incremental Behavior Change Toward Smoking Cessation, Nicotine Tob Res 2016: 20: 73-80.
