# Supplementary material for: Telephone Counseling and Messaging Guided by Mobile Profiling of Tobacco Users for Smoking Cessation: A Randomized Clinical Trial
Source: JAMA Netw Open. 2025 Mar 14;8(3):e250764. doi: 10.1001/jamanetworkopen.2025.0764 (PMC11909611; doi:10.1001/jamanetworkopen.2025.0764)
Supplement: Supplement 2. — eMethods. Specific Details eReferences eTable 1. Example of Instant Messaging Guided by mHealth Profiling via EMA eTable 2. Baseline Characteristics by Follow-Up Status at 3-Month Follow-Up (N = 459) eTable 3. Sensitivity Analysis for Primary, Secondary, and Other Outcomes eTable 4. Multivariable Linear Mixed Models for Change of Secondary, and Other Outcomes From Baseline to 6-Month Follow-Up eTable 5. Primary, Secondary, and Other Outcomes, Excluding Participants Who Exclusively Used e-Cigarettes (N = 432) eTable 6. Change of Secondary and Other Outcomes From Baseline to 6-Month Follow-Up, Excluding Participants Who Exclusively Used e-Cigarettes (N = 432) eTable 7. Process Evaluation in the Intervention Group eTable 8. Association Between EMA Completion Rate With Primary, Secondary, and Other Outcomes in all Participants (N = 459) eTable 9. Costs of Conducting the Study Including Costs for Participants’ Recruitment, Incentives, EMA App Development, and Manpower eFigure 1. Description of Triggers for Smoking Behaviors (N = 12091) eFigure 2. Subgroup Analyses for Biochemically Validated Abstinence at 3-Month Follow-Up (N = 459) eFigure 3. Subgroup Analyses for IBC-S at 3-Month Follow-Up (N = 459) [file jamanetwopen-e250764-s002.pdf]

Supplemental Online Content

Cheung YTD, Zhang MJ, Luk TT, et al. Telephone counseling and messaging guided by mobile profiling of tobacco users for smoking cessation: a randomized clinical trial. *JAMA Netw Open*. 2025;8(3)e250764. doi:10.1001/jamanetworkopen.2025.0764

eMethods. Specific Details
eReferences
eTable 1. Example of Instant Messaging Guided by mHealth Profiling via EMA
eTable 2. Baseline Characteristics by Follow-Up Status at 3-Month Follow-Up (N=459)
eTable 3. Sensitivity Analysis for Primary, Secondary, and Other Outcomes
eTable 4. Multivariable Linear Mixed Models for Change of Secondary, and Other Outcomes From Baseline to 6-Month Follow-Up
eTable 5. Primary, Secondary, and Other Outcomes, Excluding Participants Who Exclusively Used e-Cigarettes (N=432)
eTable 6. Change of Secondary and Other Outcomes From Baseline to 6-Month Follow-Up, Excluding Participants Who Exclusively Used e-Cigarettes (N=432)
eTable 7. Process Evaluation in the Intervention Group
eTable 8. Association Between EMA Completion Rate With Primary, Secondary, and Other Outcomes in all Participants (N=459)
eTable 9. Costs of Conducting the Study Including Costs for Participants' Recruitment, Incentives, EMA App Development, and Manpower
eFigure 1. Description of Triggers for Smoking Behaviors (N=12091)
eFigure 2. Subgroup Analyses for Biochemically Validated Abstinence at 3-Month Follow-Up (N=459)
eFigure 3. Subgroup Analyses for IBC-S at 3-Month Follow-Up (N=459)

This supplemental material has been provided by the authors to give readers additional information about their work.

© 2025 Cheung YTD et al. *JAMA Network Open.*

eMethods

Further details on the exclusion criteria and the salivary cotinine test

Pregnant individuals were excluded due to safety concerns with smoking cessation medications and the need for early, specialized person-to-person psychosocial interventions [1]. Individuals who had psychiatrist-diagnosed mental illness were also excluded because smoking cessation may exacerbate psychiatric symptoms and alter the pharmacokinetics of certain medications and require specialized smoking cessation interventions with close clinical monitoring [1]. The smoking status of exclusive e-cigarette users was also validated using salivary cotinine test kits, as cotinine levels are similar in regular e-cigarette users and cigarette smokers [2].

Further details on the recruitment method

Participants were recruited by advertisements via mass emails within the University of Hong Kong (HKU), social media (Instagram and Facebook), and digital media platforms (Yahoo News), and offline posters in the HKU campus.

Remotely recruitment procedures

To recruit those who did not intend to use cessation services and smoking cessation medications, our recruitment advertisement emphasized that our study was to record personal smoking features via EMA, and we would not provide intensive smoking cessation treatments and medications. Interested participants completed an online enrolment form. Potential participants' smoking status was validated by a cotinine saliva test kit, which was mailed prior to the virtual recruitment session. Eligible participants were guided to complete the online consent form and baseline questionnaire and set up the EMA app during the virtual recruitment session.

Further details on the 7-day EMA

The schedule of 5 EMAs every day for 7 days was chosen based on a meta-analysis, which indicated a balance between optimizing participant engagement and minimizing burden[3].

For each EMA, if participants failed to respond to the first notification within 5 minutes, two subsequent notifications were prompted within the next 10 minutes. The corresponding EMA would be treated as no response if these additional notifications were not answered. These data were securely sent to a HKU server.

Sleep quality was assessed in the first EMA every day, measured by: (1) "Did you experience insomnia problems last night (difficulty falling asleep, difficulty staying asleep, problems waking up too early)?" with scores ranging from 0 (none) to 4 (very severe), and (2) "How satisfied were you with your sleep pattern last night", with a score ranging from 0 (very satisfied) to 4 (very dissatisfied))?"**Further details on the mHealth profiling**

For participants who used conventional cigarettes or HTPs, daily HSI was measured by time to the first cigarette after waking up of the day and number of cigarettes the day. For exclusive e-cig users, the daily HSI was measured by time to the first e-cig after waking up of the day and times of e-cig used the day, with one 'time' defined as approximately 15 puffs or a 10-minute duration, equivalent to one conventional cigarette[4].

Further details on the nurse-led telephone counseling

An example of such advice is "Your nicotine dependence is high according to the profiling. Tobacco is in control of you. It will continuously increase your risk of cancer, heart disease and other diseases. Given your high nicotine dependence, I recommend pharmacotherapy, a proven method to reduce withdrawal

symptoms and cravings, thereby increasing your chances of successful cessation”.

Further details on biochemical validation for self-report quitters

Self-report quitters using nicotine replacement therapy (NRT) were validated by exhaled carbon monoxide only. Self-report quitters who refused face-to-face validation were only validated by a mailed cotinine saliva test kit[5]. Exclusive e-cig users at baseline who self-reported quitting at follow-ups should be validated by cotinine saliva test kits[5]. In the final analysis, validated tobacco abstinence included participants who passed either the exhaled carbon monoxide test or the salivary cotinine test, or both.

Further details on self-efficacy measurement

Self-efficacy was measured by 3 items, including perceived importance to quit on a scale of 0 (least important) to 10 (most important), perceived difficulty to quit on a scale of 0 (least difficult) to 10 (most difficult), and perceived confidence to quit on a scale of 0 (least confident) to 10 (most confident).

Further details on perceived helpfulness and satisfaction with the intervention

At 3-month follow-up, perceived helpfulness of the intervention in increasing motivation to quit, and perceived helpfulness of the intervention in successful quitting on a scale of 0 (not helpful at all), 1 (slightly helpful), 2 (somewhat helpful), to 3 (very helpful), and satisfaction with the intervention on a scale of 0 (very dissatisfied) to 4 (very satisfied), were assessed.

Further details on multivariable regressions and sensitivity analyses

Our original analysis plan included adjustment for imbalanced baseline sociodemographic characteristics in the regression models[6]. Given that we found no substantial difference in baseline sociodemographic characteristics, we also examined models adjusting for baseline characteristics that could influence outcomes (sex, age, baseline HSI, baseline readiness to quit, and baseline previous quit attempts) for primary and secondary outcomes. Two additional prespecified sensitivity analyses, including multiple imputations by chained equations to impute missing outcome data, and complete case analyses were also done for logistic and linear regressions. We did not apply sensitivity analyses in linear mixed modelss.

Further details on subgroup analyses and participants’ intervention engagement

To evaluate intervention consistency, we examined the intervention effect on the primary outcomes in subgroups of baseline characteristics. sex, age, education level, household income, nicotine dependence level (HSI), readiness to quit, previous quit attempts, and quitting self-efficacy (3 items) at baseline. To explore any potential mediation from the intervention engagement, logistic and linear regressions were used to examine the associations between the number of WhatsApp/WeChat responses by participants (documented in conversation log), the number of messages read by participants (measured by the question, “How many instant messages have you read?” with response options of “None/ A little/ Half/ Most/ All”), and EMA adherence rates with the primary outcomes in the intervention group. A third sensitivity analysis for the effectiveness of the intervention on primary and secondary outcomes by excluding 27 exclusive e-cig users was conducted.

© 2025 Cheung YTD et al. *JAMA Network Open.*

eReferences

1. Fiore MC*, et al.* Treating Tobacco Use and Dependence: 2008 Update.Clinical Practice Guideline. (U.S. Department of Health and Human Services.Public Health Service., 2008).
2. Marsot, A. & Simon, N. Nicotine and Cotinine Levels With Electronic Cigarette: A Review. *Int J Toxicol* **35**, 179-185 (2016).
3. Jones, A.*, et al.* Compliance with ecological momentary assessment protocols in substance users: A meta-analysis. *Addiction* **114**, 609-619 (2019).
4. Foulds, J.*, et al.* Development of a questionnaire for assessing dependence on electronic cigarettes among a large sample of ex-smoking E-cigarette users. *Nicotine Tobacco Research* **17**, 186-192 (2015).
5. Benowitz, N.L.*, et al.* Biochemical Verification of Tobacco Use and Abstinence: 2019 Update. *Nicotine & Tobacco Research* **22**, 1086-1097 (2019).
6. Zhang, M.J.*, et al.* Effectiveness of personalized smoking cessation intervention based on ecological momentary assessment for smokers who prefer unaided quitting: protocol for a randomized controlled trial. *Frontiers in Public Health* **11**, 1147096 (2023).
7. Chakraborty, H. & Gu, H. A Mixed Model Approach for Intent-to-Treat Analysis in Longitudinal Clinical Trials with Missing Values. in *RTI Press Methods Report Series* (RTI Press

© 2009 Research Triangle Institute. All rights reserved., Research Triangle Park (NC), 2009).

eTable 1. Example of instant messaging guided by mHealth profiling via EMA

Type	mHealth profiling	Instant messages
EMA summary	HSI	**HSI (1-2)**<br>Heaviness of Smoking Index (HSI) has a total score of 6, with higher scores indicating higher nicotine dependence.<br>Your average HSI score is _____, which signifies your level of nicotine dependence is low.<br>It is imperative that you seize this opportunity and quit smoking immediately to prevent the intensification of your nicotine addiction!<br>**HSI (3-4)**<br>Heaviness of Smoking Index (HSI) has a total score of 6, with higher scores indicating higher nicotine dependence.<br>Your average HSI score is _____, which signifies your level of nicotine dependence is moderate.<br>Your body's reliance on nicotine has deepened, the deeper your addiction, the weaker your control over smoking<br>**HSI (5-6)**<br>Heaviness of Smoking Index (HSI) has a total score of 6, with higher scores indicating higher nicotine dependence.<br>Your average HSI score is _____, which signifies your level of nicotine dependence is high.<br>You've lost control of your smoking behavior. Tobacco is now in control of you. For the sake of your health, please quit as soon as possible. You should consider seeking help from a professional.
	Individual smoking habits	According to the EMA record, it has been noted that you tend to smoke during _______ (after meals/after waking up/during breaks/when needing inspiration in work/difficulty concentrating on work).<br>You smoke _____ cigarettes per day, with the highest consumption occurring on ____ (Monday to Sunday) and the lowest on ____(Monday to Sunday). We would be interested to know why you tend to consume the most tobacco products on ____(Monday to Sunday).
	Personal triggers for smoking behavior	According to the EMA record, the top three triggers that prompt you to smoke are: ________, ________, and ________.<br>The top three triggers that induce your craving for smoking are: ________, ________, and ________.<br>The top three triggers that lead you to purchase tobacco products are: ________, ________, and ________.<br>There are indeed numerous strategies beyond smoking and buying cigarettes to cope with these triggers. Over the next nine weeks, I will share various pieces of information tailored to your current triggers, with the aim of assisting you in establishing a healthier lifestyle.
	Types of	According to the EMA record, you have reported a total of ______ instances of cravings. Of these, _____

© 2025 Cheung YTD et al. *JAMA Network Open*.

	cravings	instances were physiological cravings, _____ were psychological cravings, and ______ were social cravings.
Harmfulness of smoking	Sex and type of tobacco product used	**Male who used conventional cigarettes**<br>Detriments of Smoking: It is widely known that smoking leads to lung cancer, respiratory diseases, heart disease, and stroke. Additionally, smoking can cause erectile dysfunction and periodontal disease in men, as well as trigger various types of cancer and peripheral vascular diseases.<br>**Female who used conventional cigarettes**<br>Detriments of Smoking: Smoking is known to cause lung cancer, respiratory diseases, heart disease, and stroke. Furthermore, it can disrupt the endocrine system in women leading to early onset of menopause and increased risk of infertility. Aesthetic impacts include yellowing teeth and nails as well as accelerated skin aging. Both active smoking and exposure to secondhand smoke can affect fetal development.<br>**Male or female who used e-cigarettes/HTP**<br>As of April 30th, 2022, importing, promoting, manufacturing, selling or possessing alternative tobacco products for commercial purposes have been deemed illegal. Violators may be fined HKD 1,500 and imprisoned for six months.<br>For more information visit: https://www.smokefree.hk/page.php?id=80&lang=en
Benefits of quitting	Type of tobacco product used	**Conventional cigarettes and unwilling to quit in the nurse-led telephone counseling**<br>Benefits of Smoking Cessation: Regardless of age, quitting smoking can bring immediate and long-term health benefits. It significantly reduces the risk of disease and for those with chronic illnesses, it can delay disease progression and reduce mortality rates.<br>**Conventional cigarettes and willing to quit in the nurse-led telephone counseling**<br>Reminder: You previously mentioned your intent to reduce or quit smoking on _____ (date). It is indeed time to give yourself a chance to achieve this.<br>Benefits of Smoking Cessation: Quitting smoking can bring immediate and long-term health benefits at any age. It significantly reduces the risk of disease; for those with chronic illnesses, it can delay disease progression and reduce mortality rates.<br>**E-cigarettes/HTP and unwilling to quit in the nurse-led telephone counseling**<br>E-cigarettes/HTPs also contain harmful substances and carcinogens that can lead to various cancers, cardiovascular diseases, respiratory diseases etc., so please consider quitting them as soon as possible.<br>Although e-cigarette/HTP smoke may be odorless (or even fragrant), it contains harmful substances such as polycyclic aromatic hydrocarbons, volatile organic compounds, ultrafine particles etc. The secondhand smoke produced also affects the health of those around you.<br>E-cigarettes/HTPs are not tools for quitting smoking. Nicotine-containing HTPs and e-cigarettes prolong nicotine addiction in smokers, reducing their desire to quit smoking, even leading some users to consume two or more types of tobacco products simultaneously.<br>**E-cigarettes/HTP and willing to quit in the nurse-led telephone counseling**

© 2025 Cheung YTD et al. *JAMA Network Open.*

		E-cigarettes/HTPs also contain harmful substances and carcinogens; hence they still pose threats related to smoking-related diseases - their hazards should not be underestimated.<br>Although e-cigarette/HTP smoke may be odorless (or even fragrant), it contains harmful substances such as polycyclic aromatic hydrocarbons, volatile organic compounds, ultrafine particles etc. The secondhand smoke produced also affects the health of those around you.<br>E-cigarettes/HTPs are not tools for quitting smoking. Nicotine-containing HTPs and e-cigarettes prolong nicotine addiction in smokers reducing their desire to quit smoking - potentially leading some users to consume two or more types of tobacco products simultaneously.<br>Regardless of age, quitting smoking brings immediate and long-term health benefits. Quitting significantly reduces the risk of illness; for those with chronic conditions it helps delay disease progression while reducing mortality rates.
Managing nicotine withdrawal symptoms	HSI	**HSI (0-4) and unwilling to quit in the nurse-led telephone counseling**<br>Your nicotine dependence is not severe, and the following methods can assist you:<br>Maintain a strong will and determination to quit.<br>Set a quit date, preferably within two weeks.<br>Share your decision to quit smoking with family and friends to garner their support.<br>Remember to discard all tobacco products, ashtrays, and lighters before your quit day.<br>Try to avoid places where people smoke.<br>**HSI (0-4) and willing to quit in the nurse-led telephone counseling**<br>Your dependence on nicotine is not severe and the following methods can assist you.<br>Maintain a firm will and determination to quit.<br>You can share your decision to quit with family and friends, seeking their support.<br>Based on the quit date you set during your previous conversation with the nurse, remember to discard all tobacco products, ashtrays, and lighters before _________ (quit date).<br>Try to avoid places where people smoke.<br>• **For smokers request referral to current smoking cessation services**<br>• Quitting/reducing smoking is not easy! You previously expressed interest in trying out the smoking cessation services, which shows your strong desire to quit smoking! We have referral you to the smoking cessation services, have you visited a nurse or doctor to get medications?<br>**HSI (5-6) and not requested a referral to smoking cessation services in the nurse-led telephone counseling**<br>Reducing/quitting smoking is not easy, and your willingness to try is commendable! The chances of success are higher with assistance!<br>The most effective method of quitting smoking is a combination of medication and counseling, which can double the quit rate.

© 2025 Cheung YTD et al. *JAMA Network Open*.

		I can refer you to a free smoking cessation service, would you be interested in trying it?<br>**HSI (5-6) and requested a referral to smoking cessation services in the nurse-led telephone counseling**<br>Reducing/quitting smoking is not easy! You previously expressed interest in trying out the smoking cessation services, which shows your strong desire to quit smoking! We have referral you to the smoking cessation services, have you visited a nurse or doctor to get medications?
	Daily cigarette consumption	**Daily cigarette consumption<15 cigarettes**<br>During the process of quitting smoking, individuals may experience withdrawal symptoms such as anxiety, irritability, depression, mood instability, difficulty concentrating, insomnia, constipation, dry mouth, coughing, and increased appetite. These withdrawal symptoms typically subside gradually within two to four weeks after quitting. You can overcome the discomfort of withdrawal symptoms by implementing small tips such as drinking lemon water, eating more fruit, and taking deep breaths.<br>**Daily cigarette consumption>=15 cigarettes**<br>Let me share three effective methods for reducing smoking with you:<br>Method 1: Gradually reduce your daily smoking consumption by 25% in the first week, 50% in the first month, 75% in the second month, and completely quit by the third month.<br>Method 2: Decrease the frequency of smoking (extend the time between cigarettes).<br>Method 3: Prioritize quitting the cigarettes that are easiest to give up first (for example, the one smoked on the way home); and leave the hardest ones to quit last (for example, the one smoked first thing in the morning or after meals).
Coping strategies for personal triggers[a]	Withdrawal triggers	**Restlessness**<br>Experiencing tension or restlessness and resorting to smoking not only wastes money but also harms your health. Instead, consider engaging in deep breathing exercises.<br>The method of deep breathing: Simulate the breathing pattern of smoking, inhale air slowly for 5 seconds, hold your breath for 2 seconds, and exhale slowly for 5 seconds. Repeat the deep breathing exercise every 30 seconds, for a total of five repetitions.<br>**Fatigue**<br>Avoiding the consumption of beverages containing caffeine or alcohol can alleviate your insomnia, thereby reducing feelings of fatigue during the day.
	Emotional triggers	**Stress**<br>Desiring a cigarette when feeling stressed, it might be more beneficial to temporarily remove yourself from the stressful environment and consume some healthy snacks instead.<br>**Happiness/excitement**<br>Do you feel the urge to smoke during positive emotional states (such as happy or excited)? Reflect on what is truly important to you in your heart - the happiness and health of your family? Your personal health image? We

		hope that you can find the intrinsic motivation to quit smoking!
	Social triggers	**Participating in social gatherings**<br>In the early stage of smoking cessation, it is advisable to avoid attending social gatherings or only participate in gatherings held in smoke-free environments.<br>**Seeing family members using any tobacco products**<br>Public commitments are often more successful than private ones, so inform your family that you are quitting smoking and ask for their support. Request them to avoid smoking in your presence. If possible, invite family members who smoke to quit along with you, as doing so collectively can provide additional motivation.
	Habitual triggers	**After meal**<br>Consider replacing the habit of smoking after meals or during breaks with activities such as walking, drinking a glass of lemon water, or chewing gum.<br>**After wake up**<br>The desire to smoke upon waking is likely a withdrawal symptom due to lack of nicotine. However, rest assured! These symptoms will disappear as your body adapts, typically within two to four weeks.
Coping strategies for nicotine craving[a]	Physical craving	When experiencing a physiological craving, you can engage in the "Ten-Second Exercise to Resist Nicotine Cravings"<br>Link to the exercise: https://www.youtube.com/watch?v=mZex2Wwy3fU
	Psychological craving	Consider establishing new hobbies and interests, such as gardening, reading, playing chess, walking, and exercising, to replace smoking with activities that are beneficial to both your physical and mental health.
	Social craving	Refusing a cigarette offered by a friend in a social situation is quite normal these days. Social smoking implies that those around you will be exposed to secondhand smoke, which increases the risk of diseases such as lung cancer, heart disease, and stroke

[a]We have only listed a portion of the coping strategies for personal triggers and types of nicotine cravings.

© 2025 Cheung YTD et al. *JAMA Network Open.*

eTable 2. Baseline characteristics by follow-up status at 3-month follow-up (N=459)

	Completed follow-up (N=412)	Lost to follow-up (N=47)	P
Age, years (mean, SD)	37.1 (10.6)	32.9 (10.9)	.009
Sex			.97
Male	273 (66.3)	31 (66.0)	
Female	139 (33.7)	16 (34.0)	
Education level, n (%)			.49
Primary or below	3 (0.7)	1 (2.1)	
Secondary	191 (46.4)	24 (51.1)	
Post-secondary	218 (52.9)	22 (46.8)	
Monthly household income (HKD), n (%)[a]			.99
<30000	209 (50.9)	23 (51.1)	
30000-59999	148 (36.0)	16 (35.6)	
>=60000	54 (13.1)	6 (13.3)	
Type of tobacco product daily used, n (%)[b]			
Conventional cigarettes	372 (90.3)	44 (93.6)	.46
Heated tobacco products (HTP)	40 (9.7)	3 (6.4)	.53
Electronic cigarettes (e-cig)	71 (17.2)	13 (27.7)	.08
Nicotine dependence level (HSI score), n (%)			.34
Low (0-2)	187 (45.4)	19 (40.4)	
Moderate (3-4)	188 (45.6)	26 (55.3)	
High (5-6)	37 (9.0)	2 (4.3)	
Readiness to quit, n (%)[a]			.24
Within 30 days	53 (12.9)	10 (21.7)	
Within 6 months	52 (12.6)	6 (13.1)	
Over 6 months or not decided yet or no	307 (74.5)	30 (65.2)	
IBC-S (0-24), (mean, SD)[a]	6.1 (3.2)	5.7 (2.8)	.36
Previous quit attempt, n (%)[a]			.94
No	150 (36.4)	17 (37.0)	
Yes	262 (63.6)	29 (63.0)	
Perception of quitting (0-10), (mean, SD)			
Importance[a]	5.7 (3.0)	5.6 (3.6)	.78
Difficulty[a]	7.2 (2.7)	6.8 (2.7)	.29
Confidence[a]	4.6 (2.6)	4.8 (2.8)	.57

[a]Data were not available in all participants

[b]Some participants are daily dual users of more than one type of tobacco products

© 2025 Cheung YTD et al. *JAMA Network Open.*

eTable 3. Sensitivity analysis for primary, secondary, and other outcomes

	Completed case analysis				Multiple imputation[a]	
	Intervention group	Control group	Crude OR/β (95% CI)[b]	P-value	Crude OR/β (95% CI)[b]	P-value
**Primary outcomes**						
Biochemically validated tobacco abstinence at 3-month, n (%)	19 (9.5)	8 (3.8)	2.65 (1.13-6.20)	0.03	2.34 (1.02-5.34)	.04
IBC-score at 3-month[d], mean (SD)	8.85 (3.0)	7.66 (2.6)	1.19 (0.64-1.74)	<0.001	1.05 (0.52-1.57)	<.001
**Secondary outcomes, n (%)**						
Biochemically validated tobacco abstinence at 6-month	22 (11.4)	9 (4.6)	2.66 (1.19-5.93)	0.01	2.10 (1.01-4.33)	.046
Self-reported 7-day point prevalence abstinence at						
3-month	32 (15.9)	11 (5.2)	3.44 (1.68-7.04)	0.001	3.00 (1.50-5.98)	.002
6-month	31 (16.1)	17 (8.7)	2.00 (1.07-3.76)	0.03	1.72 (0.97-3.07)	.07
Use of smoking cessation services from baseline to						
3-month	28 (14.0)	4 (1.9)	8.42 (2.90-24.49)	<0.001	6.34 (2.27-17.69)	<.001
6-month (cumulative)	40 (18.6)	7 (3.2)	6.86 (3.00-15.70)	<0.001	5.44 (2.40-12.35)	<.001
Use of smoking cessation medications from baseline to						
3-month	36 (17.9)	7 (3.3)	6.36 (2.76-14.66)	<0.001	4.56 (2.05-10.15)	<.001
6-month (cumulative)	43 (20.0)	8 (3.7)	6.53 (2.99-14.26)	<0.001	5.15 (2.39-11.13)	<.001
**Other outcomes, n (%)**						
Quit attempts from baseline to[c]						
3-month	58 (34.3)	46 (23.0)	1.74 (1.11-2.76)	0.02	1.62 (1.04-2.51)	.03
6-month (cumulative)	68 (39.8)	56 (28.6)	1.65 (1.07-2.55)	0.02	1.64 (1.06-2.51)	.03

OR=odds ratio;β= regression coefficient

[a]Multiple imputations by chained equation models were used to impute missing data in abstinence outcomes by assuming that the data were missing at random

[b]All analyses were by logistic or linear regression

[c]Excluding participants who self-reported quitting.

© 2025 Cheung YTD et al. *JAMA Network Open.*

eTable 4. Multivariable linear mixed models for change of secondary, and other outcomes from baseline to 6-month follow-up

	Intervention group	Control group	Multivariable mixed model	
			Adjusted β (95% CI)[a]	P-value
**IBC-score, mean (SE)**				
Baseline	6.1 (0.21)	6.0 (0.20)		
3-month	8.9 (0.21)	7.7 (0.18)	1.02 (0.45-1.58)	<.001
6-month	8.6 (0.21)	7.6 (0.19)	0.94 (0.36-1.51)	.001
**Perceived importance to quit (0-10), mean (SE)**				
Baseline	6.0 (0.20)	5.4 (0.21)		
3-month	5.6 (0.19)	5.4 (0.21)	-0.43 (-0.93-0.07)	.09
6-month	5.9 (0.20)	5.6 (0.20)	-0.27 (-0.78-0.25)	.31
**Perceived difficulty to quit (0-10), mean (SE)**				
Baseline	7.2 (0.18)	7.1 (0.18)		
3-month	7.0 (0.17)	7.1 (0.18)	-0.11 (-0.53-0.31)	.60
6-month	7.0 (0.18)	7.1 (0.17)	-0.13 (-0.56-0.30)	.56
**Perceived confidence to quit (0-10), mean (SE)**				
Baseline	4.9 (0.16)	4.4 (0.18)		
3-month	5.4 (0.18)	4.3 (0.19)	0.55 (0.05-1.06)	.03
6-month	5.0 (0.21)	4.5 (0.19)	-0.03 (-0.54-0.48)	.91

β= regression coefficient

[a]Linear mixed models include sex, age, readiness to quit, nicotine dependence level (HSI), previous quit attempts, treatment group, assessment timepoint, and a treatment group-by-time interaction term as fixed effects, and participants as a random effect.

© 2025 Cheung YTD et al. *JAMA Network Open.*

eTable 5. Primary, secondary, and other outcomes, excluding participants who exclusively used e-cigarettes (N=432)

	Intervention group (N=220)	Control group (N=212)	Logistic/Linear regression			
			Crude OR /β (95% CI)[a]	P-value	Adjusted OR/β (95% CI)[b]	P-value
**Primary outcomes**						
Biochemically validated tobacco abstinence at 3-month, n (%)	16 (7.3)	6 (2.8)	2.69 (1.03-7.02)	.04	3.05 (1.14-8.16)	.03
IBC-score at 3-month, mean (SD)	8.5 (3.2)	7.7 (2.7)	0.81 (0.24-1.37)	.005	0.79 (0.25-1.32)	.004
**Secondary outcomes, n (%)**						
Biochemically validated tobacco abstinence at 6-month	19 (8.6)	8 (3.8)	2.41 (1.03-5.63)	.04	3.08 (1.25-7.61)	.02
Self-reported 7-day point prevalence abstinence at						
3-month	29 (13.2)	9 (4.3)	3.42 (1.58-7.42)	.002	3.68 (1.67-8.13)	.001
6-month	28 (12.7)	14 (6.6)	2.06 (1.05-4.04)	.04	2.46 (1.21-5.01)	.01
Use of smoking cessation services from baseline to						
3-month	27 (12.3)	4 (1.9)	7.27 (2.50-21.17)	<.001	7.88 (2.66-23.30)	<.001
6-month (cumulative)	38 (17.3)	7 (3.3)	6.11 (2.66-14.03)	<.001	6.90 (2.94-16.20)	<.001
Use of smoking cessation medications from baseline to						
3-month	34 (15.5)	6 (2.8)	6.28 (2.58-15.29)	<.001	6.85 (2.74-17.09)	<.001
6-month (cumulative)	40 (18.2)	7 (3.3)	6.51 (2.84-14.89)	<.001	7.08 (3.02-16.58)	<.001
**Other outcomes, n(%)**						
Quit attempts from baseline to[c]						
3-month	56 (29.3)	44 (21.7)	1.50 (0.95-2.37)	.08	1.54 (0.94-2.51)	.09
6-month (cumulative)	64 (35.8)	54 (27.7)	1.46 (0.945-2.25)	.09	1.45 (0.90-2.35)	.13

OR=odds ratio; β= regression coefficient

[a]All analyses were done by logistic regression or linear regression.

[b]Multivariable logistic and linear regressions adjusted for sex, age, nicotine dependence level (HSI), readiness to quit, and previous quit attempts

[c]Exclude participants who self-reported quitting.

© 2025 Cheung YTD et al. *JAMA Network Open.*

eTable 6. Change of secondary, and other outcomes from baseline to 6-month follow-up, excluding participants who exclusively used e-cigarettes (N=432)

	Crude model				Multivariable model	
	Intervention group	Control group	Crude β (95% CI)[a]	P-value	Adjusted β (95% CI)[b]	P-value
**IBC-score, mean (SE)**						
Baseline	6.11 (0.22)	6.16 (0.22)				
3-month	8.89 (0.22)	7.72 (0.19)	1.08 (0.49-1.66)	<.001	1.07 (0.48-1.55)	<.001
6-month	8.65 (0.22)	7.63 (0.19)	0.99 (0.39-1.59)	.001	0.98 (0.38-1.57)	.001
**Perceived importance to quit (0-10), mean (SE)**						
Baseline	6.05 (0.21)	5.57 (0.21)				
3-month	5.60 (0.20)	5.41 (0.22)	-0.36 (-0.88-0.16)	.17	-0.37 (-0.89-0.15)	.17
6-month	5.88 (0.20)	5.65 (0.21)	-0.25 (-0.78-0.29)	.37	-0.25 (-0.78-0.29)	.37
**Perceived difficulty to quit (0-10), mean (SE)**						
Baseline	7.22 (0.18)	7.25 (0.18)				
3-month	7.09 (0.18)	7.21 (0.18)	-0.11 (-0.55-0.32)	.61	-0.12 (-0.55-0.32)	.60
6-month	7.02 (0.19)	7.21 (0.17)	-0.11 (-0.56-0,33)	.62	-0.12 (-0.56-0.33)	.61
**Perceived confidence to quit (0-10), mean (SE)**						
Baseline	4.84 (0.16)	4.32 (0.19)				
3-month	5.35 (0.19)	4.20 (0.20)	0.61 (0.09-1.13)	.02	0.60 (0.08-1.12)	.02
6-month	4.95 (0.22)	4.38 (0.20)	0.001 (-0.53-0.53)	.996	0.0002 (-0.53-0.53)	.999

β= regression coefficient

[a]Linear mixed models include treatment group, assessment timepoint, and a treatment group-by-time interaction term as fixed effects, and participants as a random effect.

[b]Linear mixed models include sex, age, readiness to quit, nicotine dependence level (HSI), previous quit attempts, treatment group, assessment timepoint, and a treatment group-by-time interaction term as fixed effects, and participants as a random effect.

© 2025 Cheung YTD et al. *JAMA Network Open.*

eTable 7. Process evaluation in the intervention group

	Intervention group
Nurse-led telephone counseling, (N=156)[a]	
Perceived helpfulness in increasing motivation to quit, n (%)	
Not helpful at all	45 (28.9)
Slightly helpful	65 (41.7)
Moderately helpful	32 (20.5)
Very helpful	14 (9.0)
Perceived helpfulness in successful quitting, n (%)	
Not helpful at all	63 (40.4)
Slightly helpful	59 (37.8)
Moderately helpful	25 (16.0)
Very helpful	9 (5.8)
Satisfaction†	2.9 (0.6)
10-week instant messaging, (N=190)[b]	
Perceived helpfulness in increasing motivation to quit, n (%)	
Not helpful at all	45 (23.7)
Slightly helpful	91 (47.9)
Moderately helpful	41 (21.8)
Very helpful	13 (6.8)
Perceived effectiveness in successful quitting, n (%)	
Not helpful at all	61 (32.1)
Slightly helpful	84 (44.2)
Moderately helpful	38 (20.0)
Very helpful	7 (3.7)
Satisfaction†	2.8 (0.6)

[a]156 intervention group participants self-reported receiving nurse counseling at 3-month follow-up were asked for progress evaluation of nurse-led telephone counseling.

[b]190 intervention group participants self-reported receiving 10-week instant messaging at 3-month follow-up were asked for progress evaluation of 10-week instant messaging.

© 2025 Cheung YTD et al. *JAMA Network Open*.

eTable 8. Association between EMA completion rate with primary, secondary, and other outcomes in all participants (N=459)

Commented [m1]: Suggest revising to: "EMA adherence rate"

Outcomes	All participants (n=459)				Intervention group (n=231)				Control group (n=228)			
	Crude OR/β[a] (95% CI)	P-value	Adjusted OR/β[b] (95% CI)	P-value	Crude OR/Β[a] (95% CI)	P-value	Adjusted OR/β[b] (95% CI)	P-value	Crude OR/β[a] (95% CI)	P-value	Adjusted OR/β[b] (95% CI)	P-value
**Primary outcomes**												
Biochemically validated tobacco abstinence at 3-month	1.05 (1.02-1.09)	.005	1.05 (1.02-1.10)	.005	1.04 (1.01-1.08)	.04	1.04 (1.00-1.09)	.04	1.14 (1.02-1.28)	.018	1.15 (1.02-1.30)	.022
IBC-score at 3-month	0.01 (-0.01-0.02)	.377	0.01 (-0.01-0.02)	.42	0.01 (-0.01-0.03)	.23	0.01 (-0.01-0.03)	.16	0.00 (-0.01-0.02)	.857	-0.01 (-0.02-0.01)	.817
**Secondary outcomes**												
Biochemically validated tobacco abstinence at 6-month	1.04 (1.01-1.07)	.009	1.04 (1.01-1.08)	.01	1.03 (1.00-1.07)	.06	1.03 (0.99-1.07)	.06	1.13 (1.02-1.25)	.016	1.14 (1.02-1.28)	.020
IBC-score at 6-month	0.01(-0.01-0.02)	.194	0.01 (-0.01-0.02)	.13	0.01(-0.01-0.03)	.13	0.01(-0.01-0.03)	.09	0.01(-0.01-0.02)	.622	0.00(-0.01-0.02)	.644
Self-reported 7-day point prevalence abstinence at												
3-month	1.02 (1.00-1.05)	.022	1.03 (1.00-1.05)	.02	1.02 (1.00-1.05)	.057	1.02 (1.00-1.05)	.06	1.05 (0.99-1.11)	.093	1.05 (0.99-1.11)	.121
6-month	1.03 (1.01-1.05)	.012	1.03 (1.01-1.05)	.01	1.02 (1.00-1.04)	.08	1.02 (0.99-1.05)	.09	1.05 (1.00-1.10)	.038	1.06 (1.00-1.11)	.036
Use of smoking cessation services from baseline to												
3-month	1.00 (0.99-1.03)	.436	1.01 (0.99-1.02)	.49	1.02 (0.99-1.04)	.15	1.02 (0.99-1.04)	.17	0.98 (0.95-1.02)	.324	0.97 (0.93-1.01)	.135
6-month	1.01 (0.99-1.02)	.410	1.00 (0.99-1.02)	.48	1.01 (0.99-1.04)	.14	1.01 (0.99-1.02)	.15	0.99 (0.96-1.01)	.489	0.99 (0.95-1.02)	.379
Use of smoking cessation medications from baseline to												
3-month	1.02 (1.00-1.04)	.072	1.02 (0.99-1.04)	.08	1.02 (1.00-1.04)	.06	1.02 (1.00-1.04)	.045	1.02 (0.97-1.08)	.394	1.02 (0.96-1.07)	.502
6-month (cumulative)	1.02 (1.00-1.04)	.019	1.02 (1.00-1.04)	.02	1.03 (1.00-1.05)	.02	1.03 (1.01-1.05)	.01	1.03 (0.98-1.09)	.259	1.02 (0.97-1.08)	.393

© 2025 Cheung YTD et al. *JAMA Network Open.*

[a]All analyses were by logistic or linear regression

[b]Multivariable logistic and linear regressions adjusted for sex, age, HSI, readiness to quit, and previous quit attempts

[c]Exclude participants who self-reported quitting

© 2025 Cheung YTD et al. *JAMA Network Open*.

eTable 9. Costs of conducting the study including costs for participants' recruitment, incentives, EMA App development, and manpower

	Cost (US$) for intervention group	Cost (US$) for control group
1. Recruitment materials		
FB/IG	US$ 2.57 x 152 = US$ 390.64	US$ 2.57 x 146 = US$ 375.2
Google	US$ 3.35 x 5 = US$ 16.75	US$ 3.35 x 4 = US$ 13.4
Yahoo	US$ 23.56 x 22 = US$ 518.32	US$ 23.56 x 27 =US $ 636.12
Free Mass email	US$ 0 x 29 = US$ 0	US$ 0 x 28 = US$ 0
Friend	US$ 0 x 16 = US$ 0	US$ 0 x 14 = US$ 0
Poster	US$ 0.23 x 4 = US$ 0.92	US$ 0.23 x 7 = US$ 1.61
**Subtotal**	US$ **926.62**	US$ **1,026.33**
2. Coupons		
EMA	US$ 4,130	US$ 4,249
Baseline	US$ 6.44 x 231= US$ 1,533.84	US$ 6.44 x 228= US$ 1468.32
Participated biochemical validation at 3-m FU	US$ 6.44 x 21= US$ 135.24	US$ 6.44 x 8 = US$ 51.52
Participated biochemical validation at 6-m FU	US$ 6.44 x 22 = US$ 141.68	US$ 6.44 x 9 = US$ 57.96
**Subtotal**	US$ **5,940.76**	US$ **5,826.8**
3. EMA app		
	US$ 70.1 x 231= US$ 16,193.1	US$ 70.1 x 228 = US$ 15,982.8
**Subtotal**	US$ **16,193.1**	US$ **15,982.8**
4. Manpower		
Nurse-led telephone counselling	US$ 20.85 per hour x 148.75 hrs = US$ 3,101.43	US$ 0
Research assistant for recruitment	(1 hr per participant x 231) US$ 15.45 per hour = US$ 3,568.95	(1 hr per participant x 228) US$ 15.45 per hour = US$ 3,522.6
Research assistant in delivering personalized instant messaging	(30 instant messages x 5 minutes per message x 231) x 15.45 per hour = US$ 8,922.38	US$ 0
**Subtotal**	US$ **15,592.76**	US$ **3,522.6**
5. Other items		
Mobile phones for instant messaging apps	US$ 321.81	US$ 0
**Subtotal**	US$ **321.81**	US$ **0**
**Total**	US$ **38,975.05**	US$ **26,358.53**
Cost per participant	US$ 38,975.05/231= US$ **168.72**	US$ 26,358.53/228= US$ **115.61**
Cost per quitter biochemically validated tobacco abstinence at 3-m FU)	US$ 38,975.05/19 **= US$2051.3**	US$ 26,358.53/8 = **US $3294.8**
Cost per additional quitter	**(**US$ 38,975.05 - US$ 26,358.53) / (19-8) = US$ **1,147.87**	

Notes: Original costs were in Hong Kong dollars, US$1 = HK$7.8. Total cost for developing the EMA app is US$32,180.57, which amounts to US$70.1 per participant

© 2025 Cheung YTD et al. *JAMA Network Open*.

eFigure 1. Description of triggers for smoking behaviors (N=12091)

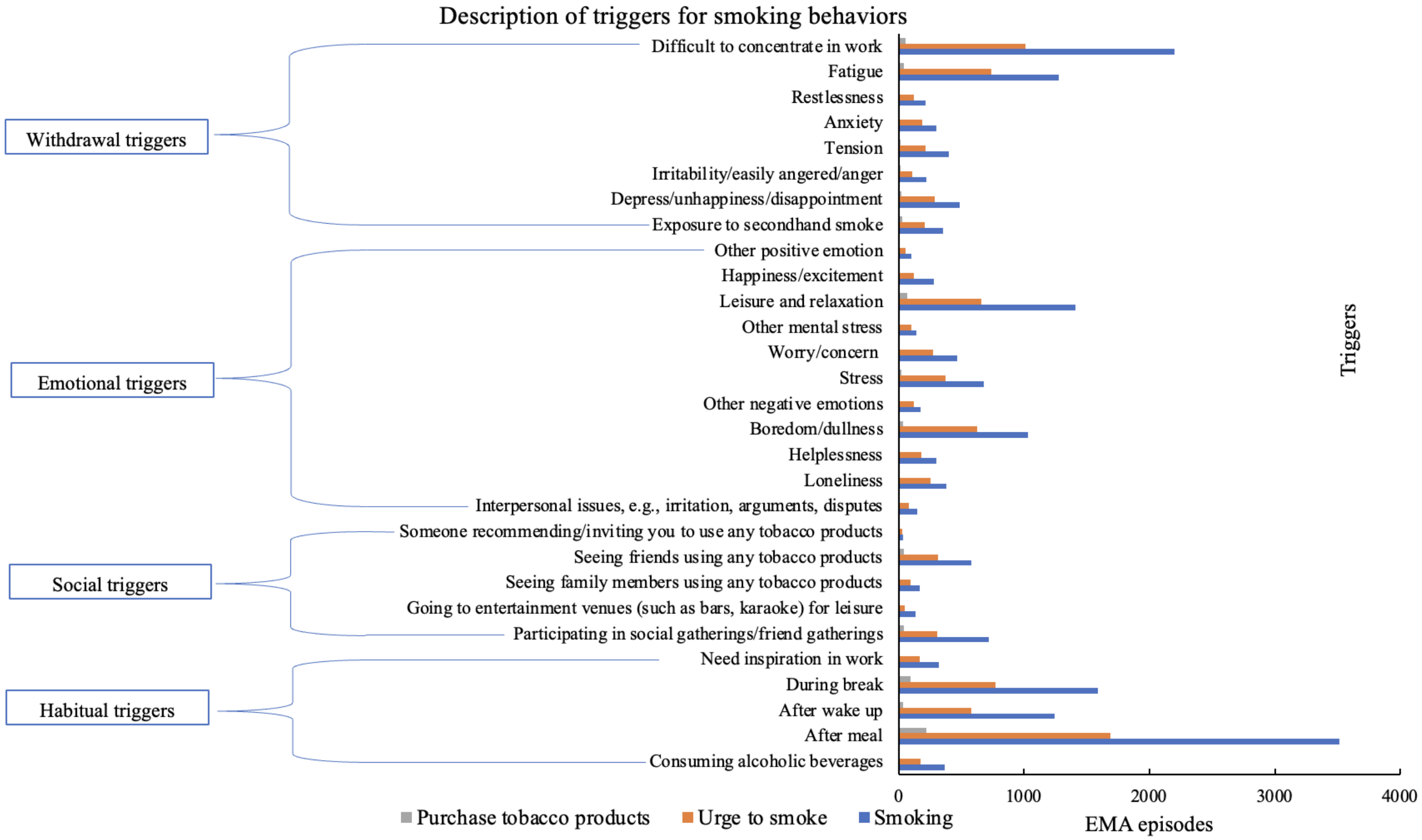

© 2025 Cheung YTD et al. *JAMA Network Open*.

eFigure 2. Subgroup analyses for biochemically validated abstinence at 3-month follow-up (N=459)

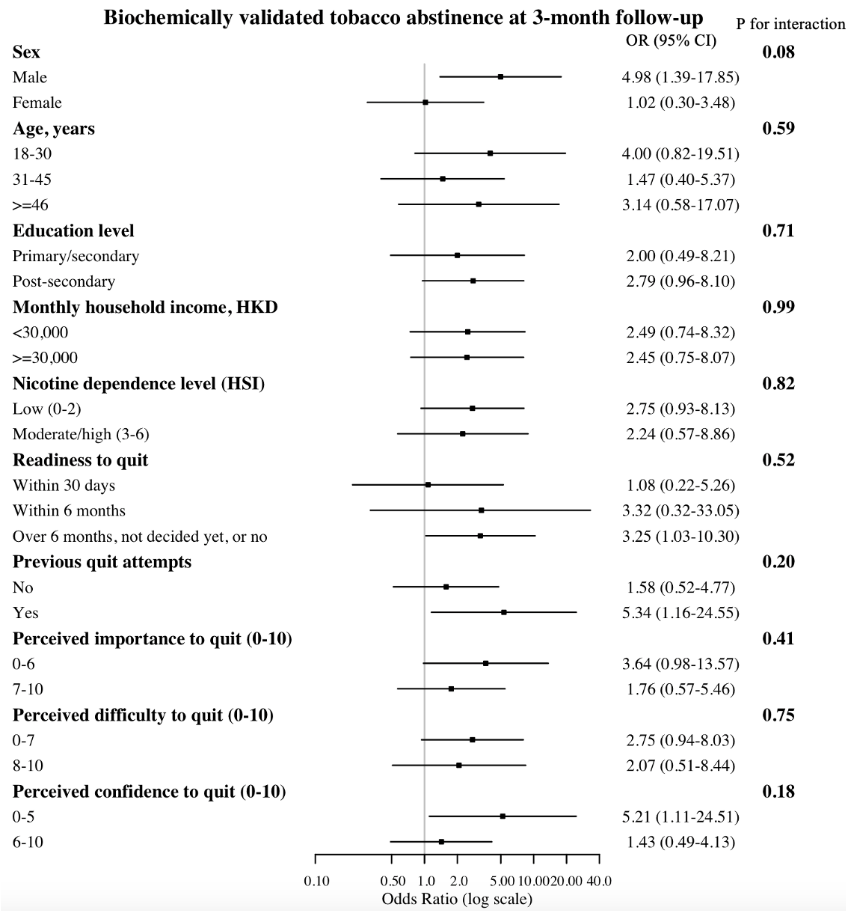

The medians of self-efficacy were used as cutoff values for subgroup analyses. The median (IQR) of importance of quitting, difficulty of quitting, and confidence of quitting were 6(4-8), 8(5-10), and 5(3-6), respectively.

© 2025 Cheung YTD et al. *JAMA Network Open.*

eFigure 3. Subgroup analyses for IBC-S at 3-month follow-up (N=459)

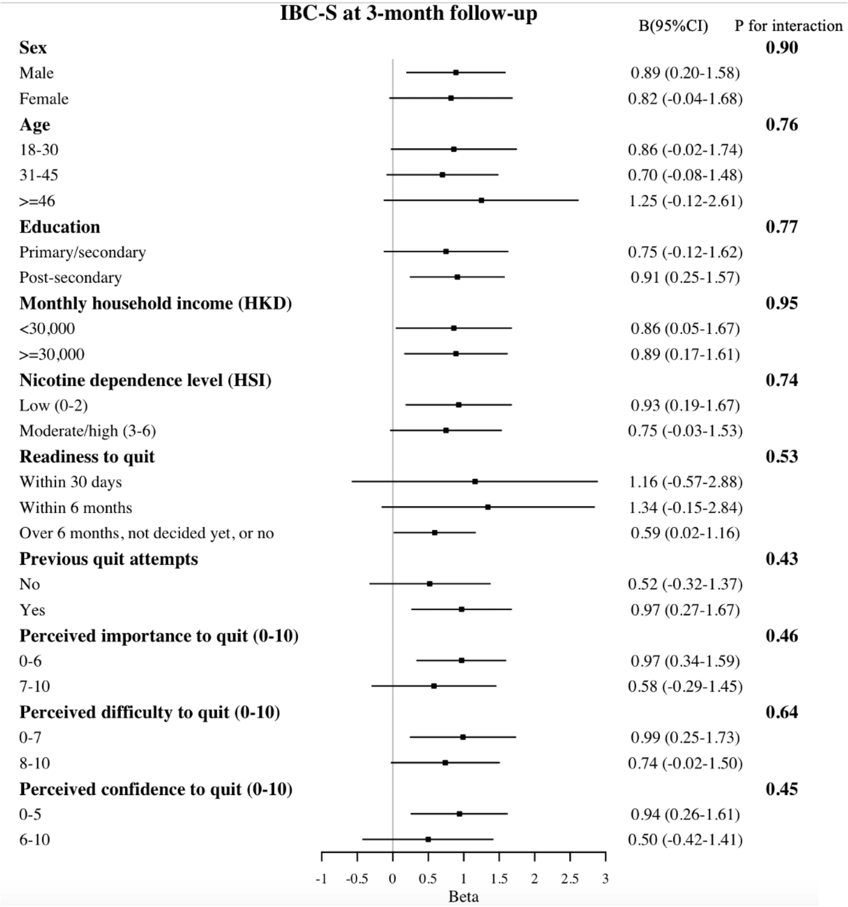

IBC-S: Incremental behavior change toward smoking cessation (score range 0-24, with a higher score indicating the more readiness and preparation in quitting)

We use the median of self-efficacy as cutoff values for subgroup analyses. The median (IQR) of importance of quitting, difficulty of quitting, and confidence of quitting were 6(4-8), 8(5-10), and 5(3-6)

© 2025 Cheung YTD et al. *JAMA Network Open.*
