# Supplementary material for: Telephone Counseling and Messaging Guided by Mobile Profiling of Tobacco Users for Smoking Cessation: A Randomized Clinical Trial
Source: JAMA Netw Open. 2025 Mar 14;8(3):e250764. doi: 10.1001/jamanetworkopen.2025.0764 (PMC11909611; doi:10.1001/jamanetworkopen.2025.0764)
Supplement: Supplement 3. — Data Sharing Statement [file jamanetwopen-e250764-s003.pdf]

Data Sharing Statement

Cheung. Telephone Counseling and Messaging Guided by Mobile Profiling of Tobacco Users for Smoking Cessation. *JAMA Netw Open*. Published March 14, 2025. doi:10.1001/jamanetworkopen.2025.0764

Data
Additional Information: ClinicalTrials.gov, NCT05212220, https://clinicaltrials.gov/study/NCT05212220?cond=NCT05212220&rank=1
Data available: Yes
Data types: Deidentified participant data
How to access data: derekcheung@hku.hk
When available: With publication

Supporting Documents
Document types: None

Additional Information
Who can access the data: researchers whose proposed use of the data has been approved
Types of analyses: for a specified purpose
Mechanisms of data availability: with investigator support, after approval of a proposal or with a signed data access agreement
